# Minimal Collective Variables for Conformational Transitions in Steered and Temperature-Accelerated MD Simulations: A T4 Lysozyme Case Study

**DOI:** 10.1021/acs.jpcb.5c01129

**Published:** 2025-05-16

**Authors:** Salsabil Abou-Hatab, Cameron F. Abrams

**Affiliations:** Department of Chemical and Biological Engineering, Drexel University, Philadelphia, Pennsylvania 19104-2816, United States

## Abstract

Conformational transitions in proteins can be difficult to observe with equilibrium molecular dynamics and challenging for enhanced sampling methods like Targeted MD when high-resolution structural data are unavailable. Low-resolution data, such as interatomic distances and angles, can serve as collective variables (CVs) to bias steered MD (SMD) simulations, but the optimal choice and number of CVs remain unclear. Here, we identify a minimal set of CVs that drive successful transitions between metastable states in T4 lysozyme. We validate them using temperature-accelerated MD (TAMD) to accelerate conformational changes in the absence of target bias. We found that CVs at both the largest and smallest scales are necessary, including interdomain hinge bending and local side-chain reorientation. A salt bridge between Arg8 and Glu64 stabilizes the closed state and must break for hinge bending, while Phe4 reorients to a hydrophobic pocket to stabilize the open state. Our results highlight the importance of selecting appropriate CVs and optimizing the steering protocol to prevent protein deformation. This work demonstrates that SMD simulations can serve as a predictive tool for understanding protein conformational changes in the absence of high-resolution structural data.

## Graphical abstract

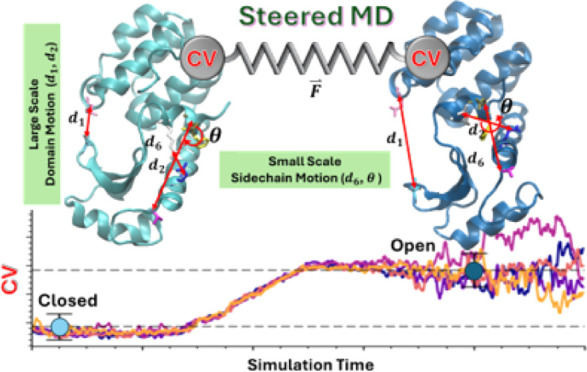

## Introduction

Many proteins execute their biological functions through well-defined conformational changes. Understanding links between structure and function for such proteins therefore requires structural information on more than just a single conformation. But determining the atomic-scale structure of any given protein in two distinct conformations does not always provide unambiguous information on the mechanism of conformational change. One can “watch” the transition between two conformational states happen using variants of all-atom molecular dynamics (MD) simulations, and when one has complete structural information on the two states, a method of choice is targeted MD (TMD). TMD uses biasing forces to minimize a system’s root mean-squared deviation relative to a known target.^1–3^

Unfortunately, it is not always easy or even possible to determine high-resolution structures of all relevant conformational states of a given protein. Some states may only be stable enough for structural characterization in the presence of other factors, like ligands or stabilizing point mutations. Some conformational states may be completely impossible to crystallize. In such cases use of TMD can be prohibitively problematic.

Here we are concerned with cases in which one conformational state is known with high resolution, and one or more distinct conformational states are characterized only by a small number of biophysical measurements. For example, methods like single-molecule FRET (smFRET)^4–9^ and cross-linking mass spectrometry (XL-MS)^10–13^ can provide constraints on certain interatomic distances in otherwise structurally uncharacterized states. If we consider these measurements as collective variables (CVs) in the context of a simulation initialized on the high-resolution structure, it is natural to ask if one biases these variables toward the values they have in a structurally uncharacterized state, can we induce a conformational change that ends with a prediction of the target conformation. Compared to the many other enhanced sampling methods that can be utilized, the simplest method to attempt such a transition is steered MD (SMD). SMD offers the advantage of simplicity and the ability to target desired values of low-resolution measurements directly.

SMD has been widely used in studies of protein–ligand binding, protein–protein interactions, unfolding pathways for small molecule drug design.^14–22^ The system is taken from an initial configuration to a final state by “pulling” one or more low-dimensional CVs, such as interatomic distances and angles using a harmonic potential. Although in theory this approach can overcome the limitations of TMD, challenges still lie in the identification of important CVs or reaction coordinates. In our case, we aim to drive a conformational change toward a target state that is only partially characterized by a limited set of low-resolution experimental measurements. These measurements, by their nature, should define key structural features of the target state and thus are natural candidates for biasing. In the absence of other detailed information, it is also reasonable to avoid biasing additional degrees of freedom that may not be directly related to the transition of interest. While using more CVs could potentially enhance sampling, doing so without justification risks introducing bias that might prevent convergence to the correct final state. Therefore, one would generally like to know the lowest dimensionality required to uncover the mechanisms that drive the system from one state to another, and thus the particular variables that need to be biased in SMD to guarantee that a conformational transition ends in the correct state. By identifying the minimal number of CVs that are empirically linked to the conformational change we would thus minimize unnecessary assumptions about the transition pathway and accurately and efficiently guide the system between metastable states using SMD.

In cases where an explicit target bias is absent or when the system’s transition pathway is not well-characterized, a more sophisticated approach is required. Temperature-Accelerated Molecular Dynamics (TAMD) has emerged as a powerful method for exploring protein conformational landscapes without relying on a target bias.^23–30^ In this method, A fictitious particle of mass *m* is introduced and coupled to CV of interest using a harmonic spring. Under the condition of adiabatic separation of variables, the fictitious particle is accelerated at a high temperature while the rest of the system evolves simultaneously at a physiological temperature. This allows the system to overcome high-energy barriers in the CV space and sample otherwise inaccessible regions. While TAMD enables broad exploration of conformational states, its stochastic nature and reliance on temperature-driven fluctuations make it challenging to precisely guide transitions toward a known structural outcome. This limitation underscores the need for methods like SMD, which applies direct mechanical control to drive transitions along experimentally constrained pathways. Moreover, the ability of TAMD simulation to facilitate a conformation change reliably depends greatly on the choice of meaningful CVs. For this reason, we use TAMD as a means to validation tool for the CVs screened through SMD. In this study, we leverage SMD to predict conformational transitions between metastable states using low-resolution data, aiming to establish a more controlled and systematic approach to protein structure prediction.

While SMD provides a direct means of inducing conformational transitions, its reliability in predicting structural changes and guiding transitions between metastable states remains uncertain. In this study we address this by investigating protein conformational transition between two metastable states using low resolution data and SMD simulation. This can lead to using SMD as a tool that can guide the prediction of protein structures where experiments provide only low-resolution information. Our testbed molecule is bacterial enzyme T4 lysozyme (T4L). Many bacterial enzymes are allosteric proteins that are capable of transitioning between different stable states by the interaction between residues at distant regions in the protein.^9,31,32^ T4L is a well-studied bacterial enzyme of medium size (164 residues) with two resolved metastable states, making it an attractive system to use as a case study.^9,31–42^ T4L plays a vital role in breaking down bacterial cell walls by catalyzing the cleavage of glycosidic bonds in saccharides.^9,31,35,36,42^

T4L has two domains. The N-domain is made up of the α_1_ helix bridged by a β-sheet to the α_2_ helix, connected by flexible loops, as shown in Figure 1. The C-domain acts as a nearly rigid body composed of an α-helix bundle (α_4_ to α_10_), which is connected to the N-domain by a long α helix (α_3_).^33–35,38^ T4 lysozyme is known to undergo hinge-bending motions between its N-terminal and C-terminal domains, even in the absence of ligands or substrates, as demonstrated by crystallographic, NMR, and single-molecule studies.^31,34–38^ These motions are intrinsic to the protein’s structure and can be observed under physiological conditions without external triggers. Nonetheless, environmental factors such as substrate binding,^9,37^ pH,^42^ and mutations^9,32^ can shift the conformational equilibrium or stabilize particular states. Both NMR^38^ and EPR^37^ studies provide clear evidence for distinct “closed” and “open” states connected by bending about a hinge linking the two domains. The time scale for this transition of T4L from closed to opened state was estimate at about 15 μs using sm-FRET.^36^ These two conformational states were also resolved through high-resolution solution-state X-ray crystallography.^33,34^ This large-scale domain motion was later captured with long MD simulations which required significantly large amounts of time and computational power.^32,39–42^

**Figure 1:**
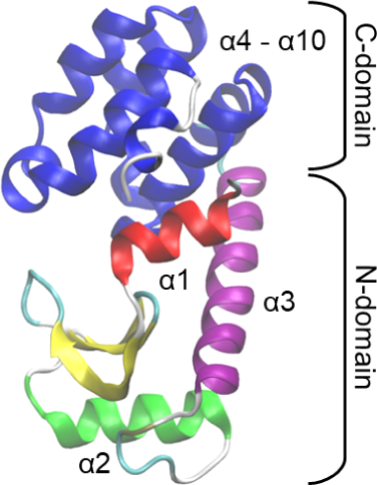
Illustration of the bacterial T4 lysozyme structure, highlighting the N-terminal domain with two short alpha-helices (α_1_, α_2_) linked to a beta-sheet, and the C-terminal domain formed by an α-helical bundle (α_4_– α_10_). The two domains are connected by a long α helix (α_3_).

In this work we explore capturing this conformational transition using relatively short SMD simulations with a few target CV values. By exploring the effects of different CVs and simulation parameters, we aim to provide insights into how SMD can predict protein conformational changes and potentially aid in cases where experimental methods struggle to fully resolve protein structures. We will first introduce the methods where we use classical MD and SMD simulations which include details on the selection of CVs, simulation protocols, and parameters used to capture the conformational transitions of T4L. We will then discuss our findings from these simulations, focusing on the effects of varying CVs, steering forces, and simulation durations. We will highlight how different conditions impacted the successful transition between the closed and open states, as well as the challenges of preventing protein deformation. We will then demonstrate how we evaluate the ability of CVs suggested by SMD to successfully induce a conformational transition without biasing them toward a target using TAMD simulations. Finally, we conclude by summarizing the key insights and the broader applicability of SMD simulations and low resolution data for modeling protein conformational transitions.

## Methods

### Equilibrium MD Simulations

We first establish reference structures of the closed and open states of T4L based on available crystal structures. Wild-type (WT) T4L in the closed state is represented by PDB entry 2LZM.^34^ PDB entry 150L^33^ is the open state of the M6I mutant missing C-terminal residues 163 and 164. To make these two systems congruent, we built a WT open reference state from PDB entry 150L by reverting the mutation at position 6.

Each of these reference states were used to initialize five independent equilibrium MD simulations. The initializations involved adding hydrogens, steepest-descent minimization, vacuum MD simulation at constant temperature of 300 K, solvation with SPC/E waters and chloride counterions, an NVT MD simulation at 300 K for 1 ns with position restraints on the protein, 100 ns at constant pressure of 1 bar and temperature of 300 K to ensure density equilibration, followed at last by a 300 ns NPT production MD simulation. Temperature was controlled using the velocity-rescaling modified Berendsen thermostat,^43^ with a time constant of 0.1 ps, and pressure was controlled using the Parrinello–Rahman barostat,^44,45^ with a time constant of 2.0 ps. Nonbonded interactions were cut off at 1 nm, and the Particle-Mesh Ewald method with a grid spacing of 0.1 nm and an interpolation order of 4 was used for electrostatics. Periodic boundary conditions were held in all directions. A time-step of 2 fs was used in all MD simulations. All simulations used the OPLS-AA/L force field^46,47^ and were conducted using Gromacs v. 2022.3.^48^

### Collective Variables

The set of CVs we consider here comprise 10 distinct interatomic distances and one side-chain dihedral angle. These are depicted on both the closed and open T4L conformations in Figure 2 with detailed CV definitions in the caption. The CVs are also listed in Table 1 in the Results and Discussion section along with the values they display in the relevant crystal structures. As we describe in the Results and Discussion, each of these variables have clear and distinct basins of high probability in each of the closed and open states of T4L.

**Figure 2:**
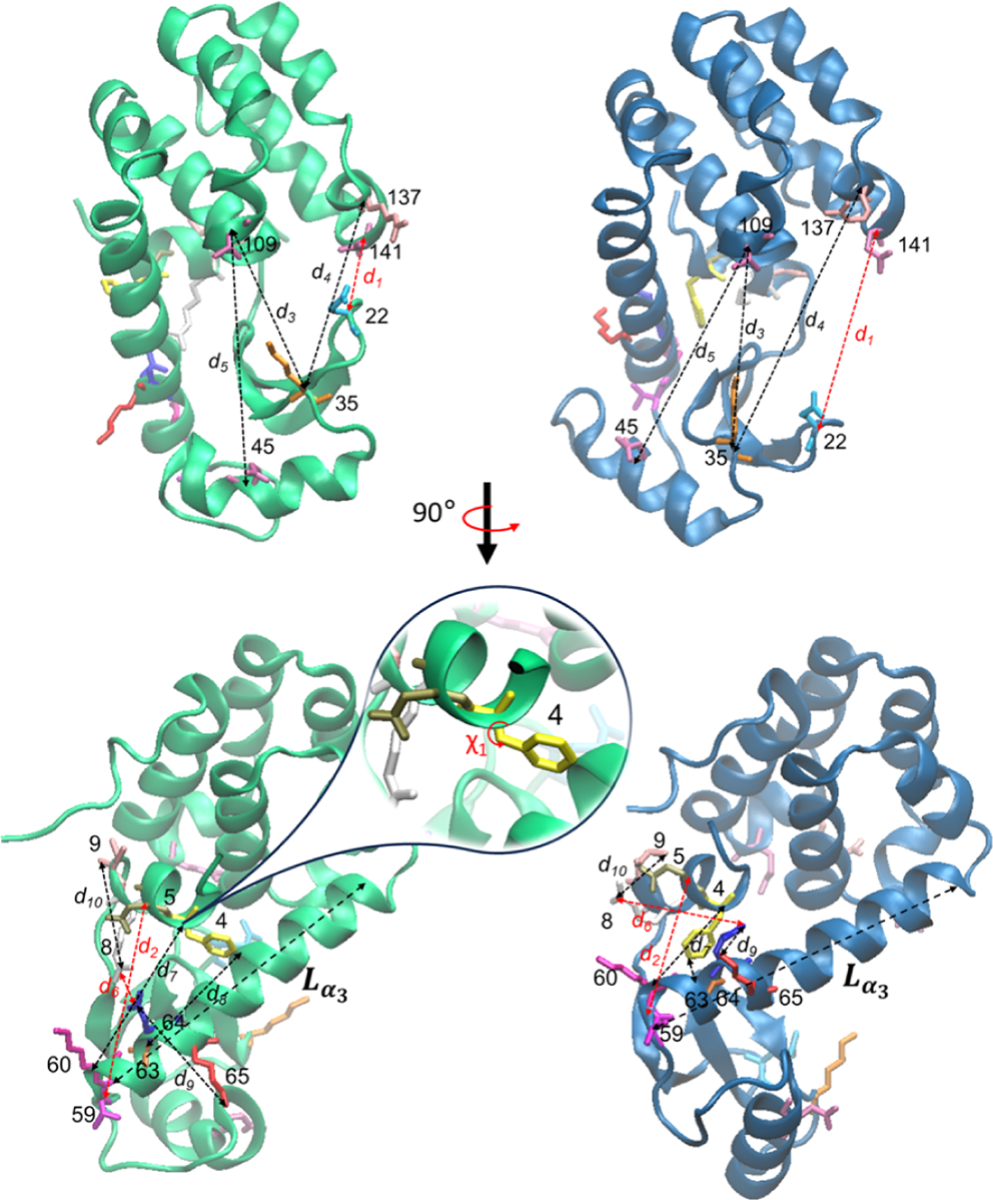
Depiction of the CVs interatomic distances top: *d*_1_ (Glu22CA and Gln141CA), *d*_3_ (Lys35CA and Thr109CA), *d*_4_ (Lys35CA and Arg137CA) *d*_5_ (GLU45CA and THR109CA), and bottom rotating the molecule by 90°: *d*_2_ (Glu5CA and Thr59CA), *d*_6_ (Arg8CZ and Glu64CD), *d*_7_ (Phe4CA and Lys60CA), *d*_8_ (Phe4CZ and Ala63CA), *d*_9_ (Arg8CZ and Ile9CB), *d*_10_ (Glu64CD and Lys65NZ), and torsion angle χ_1_ of Phe4 in the closed (cyan) and opened (blue) states. Distance between center of mass of N- and C- terminal residues of the α_3_ helix denoted $L_{\alpha_{3}}$ . Wild-type closed and open state structures shown here are equilibrium initial conditions used in the SMD simulations. In red are CVs which were biased in the SMD simulations.

**Table 1:** Values Adopted by CVs in the Closed and Open States of T4L^a^

			Closed			Open		
CV	atom 1	atom 2	MD	xtal	expt	MD	xtal	expt
*d*_1_	Glu22CA	Gln141CA	0.93 ± 0.23	0.90	—	1.92 ± 0.29	1.73	—
*d*_2_	Glu5CA	Thr59CA	1.91 ± 0.11	1.87	—	1.46 ± 0.08	1.48	—
*d*_3_	Lys35CA	Thr109CA	1.51 ± 0.19	1.61	1.61	2.00 ± 0.22	1.93	1.76
*d*_4_	Lys35CA	Arg137CA	1.81 ± 0.16	1.82	1.70	2.67 ± 0.24	2.66	2.53
*d*_5_	Glu45CA	Thr109CA	2.18 ± 0.17	2.20	—	2.74 ± 0.19	2.59	—
*d*_6_	Arg8CZ	Glu64CD	0.41 ± 0.06	1.02	—	1.04 ± 0.18	1.08	—
*d*_7_	Phe4CA	Lys60CA	1.52 ± 0.10	1.45	1.45	1.00 ± 0.06	1.04	1.05
*d*_8_	Phe4CZ	Ala63CA	1.36 ± 0.17	1.22	—	0.48 ± 0.05	0.49	—
*d*_9_	Arg8CZ	Ile9CB	0.96 ± 0.09	0.79	—	0.71 ± 0.15	0.85	—
*d*_10_	Glu64CD	Lys65NZ	1.01 ± 0.13	0.96	—	0.71 ± 0.22	0.72	—
χ_1_	Phe4		66.40 ± 27.20	65.67	—	176.10 ± 8.60	175.80	—

a All distances are reported in nm and angles in degrees. “MD” refers to the equilibrium MD simulations described in Results and Discussion section; “xtal” to values in the closed- (PDB 2LZM^34^) and open-state (PDB 150L^33^) crystal structures; and “expt” to values from the fluorescence experiments of Yirdaw and Mchaourab.^36^

*d*_1_ is the C_α_–C_α_ distance between residues of Glu22 and Gln141, which lie on the tip of the two loops connecting the α_1_ helix to the β-sheet within the N-domain and the α-helix bundle of the C-domain, respectively. This variable is associated with the hinge domain motion in the protein. *d*_2_ is the C_α_–C_α_ distance between residues Glu5 at the center of the α_1_ helix and Thr59 on the N-terminus of the α_3_ helix. The variation in the interatomic displacement of this variable is meant to mimic the bending motion of the system. *d*_3_ is the interatomic distance between Lys35CA which sits in the center of the loop connecting the β-sheet to α_2_ and the Thr109CA atom within the helical bundle adjacent to the cleft. *d*_4_ is the distance between Lys35CA and Arg137CA which neighbors Gln141 at the cleft. *d*_5_ is the C_α_–C_α_ distance between GLU45 which lies at the center of the alpha-2 helix and THR109.

In addition, although not seen in the crystal structures, our equilibrium MD simulations revealed that a salt-bridge between Arg8 and Glu64 exists in the closed state, but not in the open state. We denote the distance between CZ of Arg8 and CD of Glu64 as *d*_6_. Two other interatomic distances were used to characterize the salt-bridge: *d*_9_ (Arg8CZ and Ile9CB) and *d*_10_ (Glu64CD and Lys65NZ). The χ_1_ side-chain torsion angle of Phe4 is also crucial for distinguishing between the two states: in the WT closed 2LZM structure, the Phe4 side-chain is exposed, whereas in the open state it is buried in a hydrophobic region or sandwiched between α_1_ and α_3_ helices. As it is the only angle which we are examining we will refer to the χ_1_ of Phe4 as simply χ_1_. The distance between CZ atom of Phe4 and the CA of Ala63, denoted *d*_8_, can potentially capture the motion of both *d*_2_ and χ_1_ simultaneously and was also explored. The distance between the center of mass of the four N- and C-terminal residues of the α_3_ helix is denoted $L_{\alpha_{3}}$ . All steered variables are depicted in Figure 2 in red.

### Steered MD Simulations

The objective of the SMD simulations is to induce a conformational transition, either from closed to open or from open to closed. SMD uses external forces on a small number of CVs, and, if successful, the target state to which it drives the system remains metastable in standard MD after all biasing restraints are turned off. We developed a set of SMD simulations to determine the smallest possible set of CV’s needed to guarantee a successful transition. First, we characterized each conformation in terms of the mean and standard deviation of each CV by directly sampling five independent 300 ns MD simulations for each conformation. This provided characteristic values for each CV in both the open and closed states. Generally, steering was performed in four stages of various durations: “ramp-up”, in which the force constants are increased linearly to specified values while the CVs are restrained at their characteristic closed-state values; “steer”, in which the steering of the CVs from their closed-state characteristic values to their open-state characteristic values is performed; “ramp-down”, in which the force constants are ramped down to zero with CVs restrained at their open-state characteristic values; and finally, “free”, in which no restraints are used. A simulation is “successful” if all CV’s remain near their open-state characteristic values during the free stage. Harmonic potentials applied to distance-based CVs mostly used force constants of 500 kJ/mol·nm^2^, although we explored smaller values in some simulation sets. Those applied to angles used 1000 kJ/mol·rad^2^. Force constants were benchmarked when all four CVs were biased simultaneously to evaluate the amount of strain being applied to the secondary structure of the protein.

We systematically explored several different biasing regimes to arrive at a minimal set of CVs required to achieve reliably successful transitions. These regimes included not only changing the numbers and identities of CVs but also the durations of the four steering phases and the spring constants.

### Temperature Accelerated MD Simulations

TAMD simulations were conducted to evaluate the ability of sets of CVs used in SMD simulations to induce conformational transition without the influence of a target bias.^30,49^ CVs of interest were each coupled to a fictitious particle via a spring constant κ and evolved using Langevin dynamics at a high fictitious temperature (*T*_*f*_). The system’s dynamics and stability were regulated by two key parameters: relaxation time (τ) and fictitious friction (γ). The remainder of the system evolved at a temperature of 300 K under the same conditions described in the Equilibrium MD Simulations section. These four TAMD parameters were systematically varied until conformational transition was achieved. Five replicas of each TAMD simulation set were run for 100 ns with a time step of 2 fs. All SMD and TAMD simulations were performed with Gromacs v. 2022.3 ^48^ patched with Plumed v. 2.8.1.^49^

## Results and Discussion

### Equilibrium MD Simulations

Table 1 reports the mean and standard deviation for all 11 CV’s extracted from the closed- and open-state equilibrium MD simulations, along with their values in the relevant crystal structures and, in a few cases, measured by sm-FRET.^36^

In the closed state, the distance between Glu22CA and Gln141CA (*d*_1_) is 0.93 nm whereas in the open state it is f 1.92 nm. Large fluctuations in this variable are found in both closed and open states where the standard deviation from their means are 0.23 and 0.29 nm, respectively. *d*_3_, *d*_4_, and *d*_5_ are also found to have distinguishably greater values in the open vs closed state. In contrast, *d*_2_ is 1.91 nm in the closed state and 1.46 nm in the open state, and *d*_7_ is 1.52 nm in the open state and 1.00 nm in the closed state. The residues in this region are more crowded relative to residues related to variables associated with the hinge motion such as *d*_1_, *d*_3_, *d*_4_, and *d*_5_, and thus more steric interactions that constraints the bending motion demonstrated by small standard deviations in the *d*_2_ and *d*_7_ variables are prominent.

Our MD simulation results are in good agreement with experimentally observed residue-pair distances in the X-ray crystal structures, with the exception of *d*_6_ for the closed state. In the 2LZM crystal structure, *d*_6_ is 1.02 nm. In our MD simulation, a salt bridge between Arg8CZ and Glu64CD quickly forms and remains intact at an average distance of 0.41 nm in all simulations. *d*_6_ is thus a unique and possibly overlooked mechanistic detail crucial to the transition between the states. The equilibrium MD simulation results are also in good agreement with low-resolution experimental data from sm-FRET study that measured the conformational dynamics of T4L in solution.^36^

In the closed state, the side-chain of Phe4 is solvent exposed and shows significant fluctuation from a mean of 66.4° by 27.2°. In the open state, the aromatic side-chain is buried in a hydrophobic pocket, sandwiched between α_1_ and α_3_ at a χ_1_ average angle of 176.1°, where its motion is restricted to a standard deviation of 8.6°.

Empirical probability densities for *d*_1_, *d*_2_, *d*_6_, and χ_1_ are shown in Figure 3 for both the closed and open states. Generally, closed-state distributions are multimodal, with peak positions that do not closely match the overall averages reported in Table 1. The open state generally displays single-modal distributions that, in the case of distances, are somewhat broader than those of the closed state. The Phe4 χ_1_ is different: its distribution in the closed state, where it is solvent-exposed, is broader than its distribution in the open state, where it is buried in a hydrophobic pocket. We also note very infrequent but noticeable excursions of χ_1_ in the closed state to its open-state value, suggesting that sampling of this value of χ_1_ is spontaneous and not induced by the conformational change to the open state.

**Figure 3:**
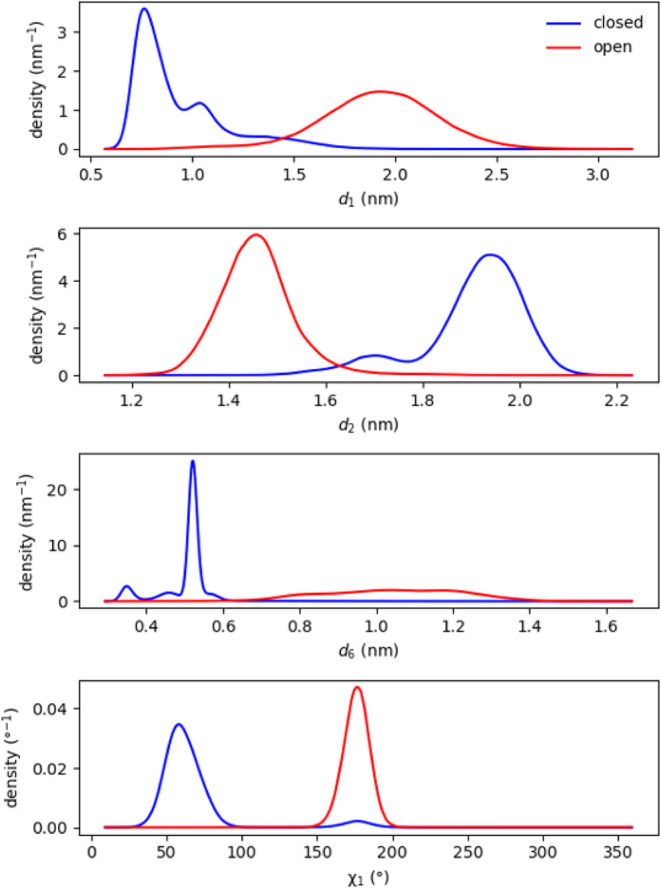
Probability density distributions of interatomic distance variables *d*_1_, *d*_2_, *d*_6_, and torsion angle χ_1_ of Phe4 for the closed and open state of T4L extracted from equilibrium MD simulations.

### Steered MD Simulations

#### The Closed-to-Open Transition

Table 2 summarizes all SMD simulations where the closed-to-open transition was attempted. Each set comprises five independent replica simulations. Each row in the table indicates the CVs biased in each set, the schedule by which the bias and steering were performed, and the number of successful transitions. The schedules are indicated by Roman numerals that refer to profiles shown in Figure 4, which specifies the durations in ns of each stage, as described in the Methods: ramp-up, steer, ramp-down, and free. For example, Schedule I devotes 25 ns to each stage.

**Table 2:** Summary of the 18 Sets of Five SMD Simulations for the Closed-To-Open Transition of T4L^a^

Set Name	CV:κ					Schedule	Result
1	*d*_1_:500					I	0
2	*d*_2_:500					I	0
12	*d*_1_:500				*d*_2_:500	I	0
12χ	*d*_1_:500	*d*_2_:500	χ_1_:1000			I	0
126	*d*_1_:500	*d*_2_:500	*d*_6_:500			I	0
126χ	*d*_1_:500	*d*_2_:500	*d*_6_:500	χ_1_:1000		I	3
186	*d*_1_:500	*d*_8_:500	*d*_6_:500			I	0
129χ	*d*_1_:500	*d*_2_:500	*d*_9_:500	χ_1_:1000		I	1
129-10χ	*d*_1_:500	*d*_2_:500	*d*_9_:500	*d*_10_:500	χ_1_:1000	I	1
126χ-(F1)	*d*_1_:200	*d*_2_:100	*d*_6_:50	χ_1_:1000		I	0
126χ-(F2)	*d*_1_:20	*d*_2_:10	*d*_6_:5	χ_1_:1000		I	0
126χ-(S1)	*d*_1_:500	*d*_2_:500	*d*_6_:500	χ_1_:1000		II	2
126χ-(S2)	*d*_1_:500	*d*_2_:500	*d*_6_:500	χ_1_:1000		III	2
126χ-(S3)	*d*_1_:500	*d*_2_:500	*d*_6_:500	χ_1_:1000		IV	1
6-12χ	*d*_1_:500	*d*_2_:500	*d*_6_:500	χ_1_:1000		*	0
$126\alpha_{3}\chi$ -(a)	*d*_1_:500	*d*_2_:500	*d*_6_:500	χ_1_:1000	$L_{\alpha_{3}}$ :500	V	1
$126\alpha_{3}\chi$ -(b)	*d*_1_:500	*d*_2_:500	*d*_6_:500	χ_1_:1000	$L_{\alpha_{3}}$ :500	II	0
$126\alpha_{3}\chi$ -(c)	*d*_1_:500	*d*_2_:500	*d*_6_:500	χ_1_:1000	$L_{\alpha_{3}}$ :500	VI	2
$126\alpha_{3}\chi$ -(d)	*d*_1_:500	*d*_2_:500	*d*_6_:500	χ_1_:1000	$L_{\alpha_{3}}$ :500	I	5

a Each set biases between 1 and 5 CVs using harmonic springs with spring constants indicated after the colon next to each CV name. The schedule refers to how the harmonic forces are turned on, used, and then turned off, and are illustrated graphically in Figure 4. (Type “*” denotes a custom schedule in which the CVs were not treated simultaneously and is described in the text.) The result is the number of successful steers in each set. The force constant, κ, is reported in kJ/mol·nm^2^ for distances di and kJ/mol·rad^2^ for torsion angle χ_1_. refers to the length the α_3_ helix, measured as the distance between the centers of mass of the four N-terminal residues to the center of mass of the four C-terminal residues.

**Figure 4:**
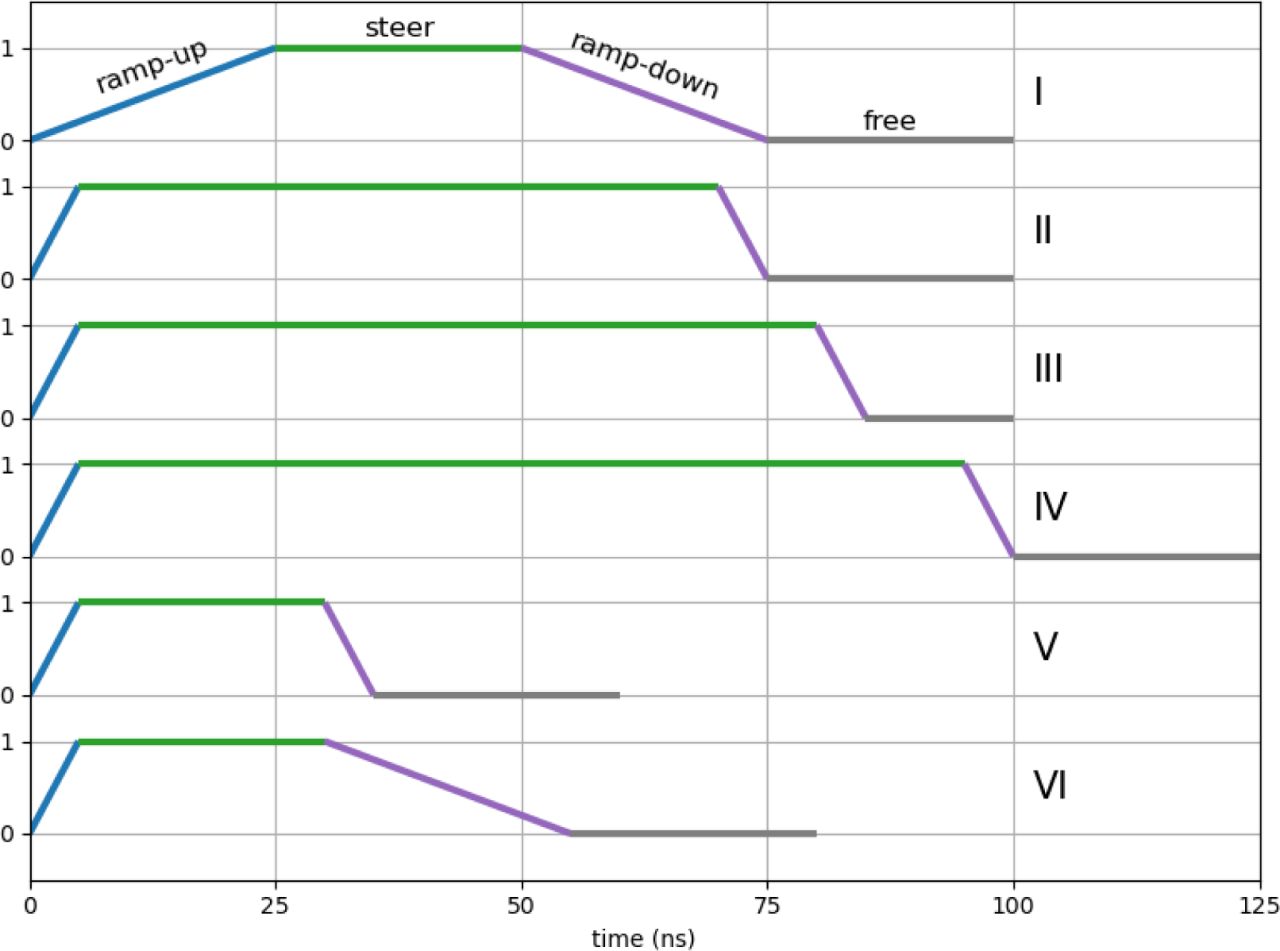
Six distinct schedules for ramping up, steering, ramping down, and freeing all spring constants in the SMD simulations.

Steering only the largest-scale CV, *d*_1_, alone is not sufficient to drive the closed-to-open transition. Figure 5A shows traces of *d*_1_, *d*_2_, *d*_6_, and χ_1_ for the five SMD simulations in which only *d*_1_ is steered. Clearly, none of the other variables were able to even sample their characteristic open-state values, apart from one instance of χ_1_. When *d*_2_ alone is steered, we observe no successful transitions, as shown in Figure 5B. Steering both *d*_1_ and *d*_2_ simultaneously resulted in no successful transitions (Figure S1), and steering χ_1_ with *d*_1_ and *d*_2_ also resulted in no successful transitions (Figure S2). A similar result was observed in the 126 set, where steering *d*_6_ alongside *d*_1_ and *d*_2_ forced the salt bridge open (Figure S3), but the side-chain of Phe4 did not rotate spontaneously into the pocket. However, we observed three out of five successful transitions when all four of *d*_1_, *d*_2_, *d*_6_, and χ_1_ were steered simultaneously. Thus, although steering the two largest scale CVs, *d*_1_ and *d*_2_, primarily drive the large-scale domain motion for the closed-to-open transition, small changes in the side-chains relative positions and rotameric states are likely responsible for the stability of the two conformations.

**Figure 5:**
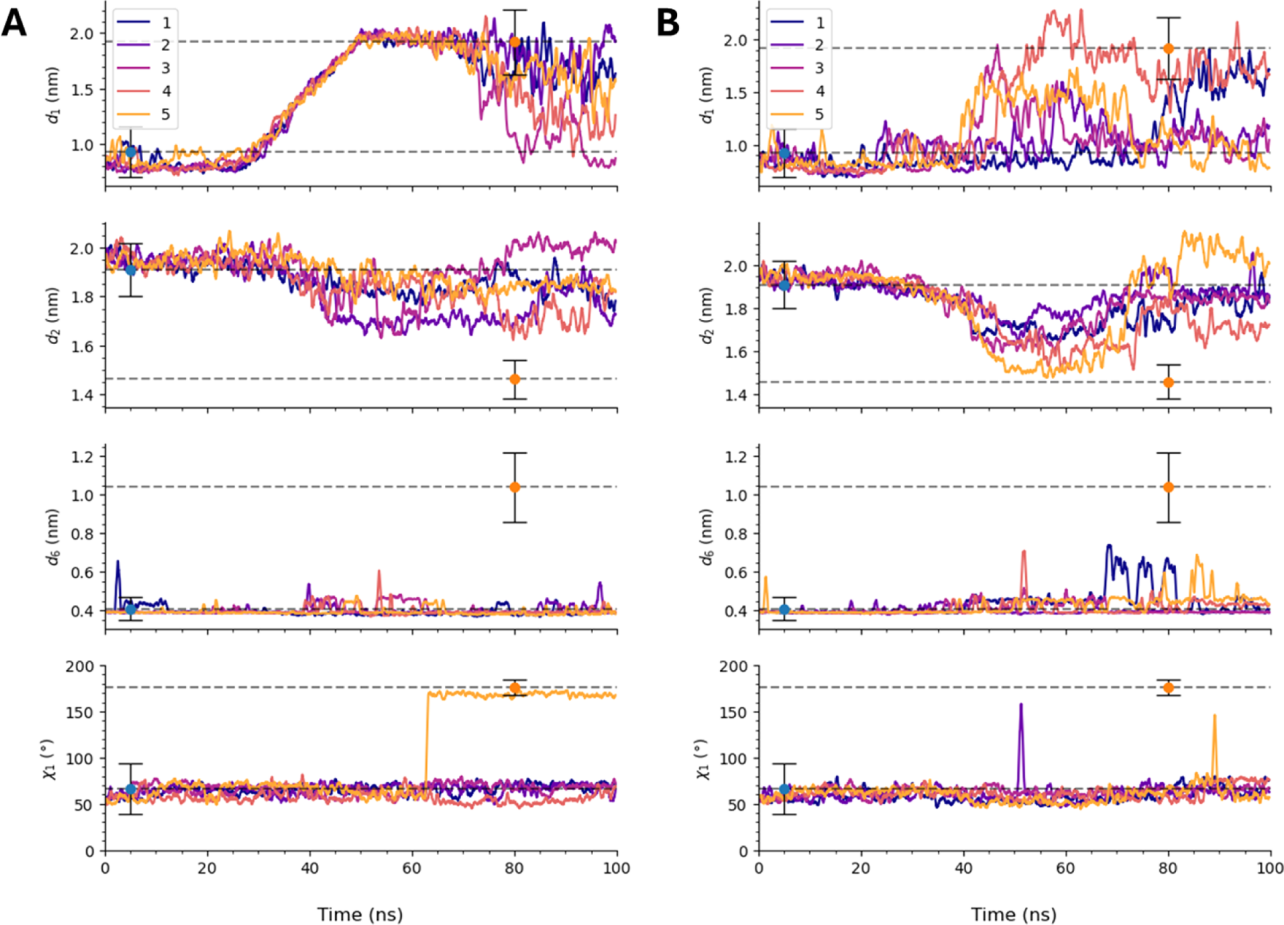
CVs *d*_1_, *d*_2_, *d*_6_, and χ_1_ vs time during SMD simulations. (A) Set 1, in which only *d*_1_ was steered. (B) Set 2, in which only *d*_2_ was steered. In both panels, dashed horizontal lines marked with blue points indicate average values of each CV in the closed state, and lines marked with orange points indicate average values of each CV in the open state. Error bars on those indicator points report the standard deviation of each CV in each state. The raw data was smoothed using a rolling function with a window size of 51.

This is exemplified by comparing Sets 12χ and 126. Placing the Phe4 side-chain into its buried pocket characteristic of the open state requires its χ_1_ to transition from about 50° to 175° while access to the pocket itself is blocked by the salt bridge between Arg8 and Glu64, which is represented by the CV *d*_6_. Set 12χ shows that, although all of *d*_1_, *d*_2_, and χ_1_ are steered, the Arg8-Glu64 salt bridge remains intact, and Phe4 does not enter its open-state pocket. When including an explicit breakage of this salt bridge by additionally steering *d*_6_, Phe4 does in fact successfully place itself in the pocket three times (Figure S15). This SMD set indicates that forcing the salt bridge open is essential for Phe4 to access and rotate into the target site. However, in the two unsuccessful steers, we found that the α_3_ helix breaks at distinct positions along the its length and at different steps of the steering. We speculate that this occurs because biasing *d*_2_ and *d*_6_ simultaneously means that we are pulling Thr59CA and Glu64CZ, which both lie at the N-terminus of α_3_, in opposite directions of one another, respectively.

In the next conceptual block of SMD sets in Table 2 we specifically address preventing α_3_ breakage by not using *d*_6_ and *d*_2_ together, and replacing one or both with other CVs. The CV *d*_8_ measures the distance from Phe4 to Ala63, with low values indicating Phe4’s side-chain docked in the open-state pocket. Steering *d*_1_, *d*_8_, and *d*_6_ together, however, both resulted in no successful transitions and broke α_3_ multiple times. CV *d*_9_ drives breakage of the Arg8-Glu64 salt bridge by pulling the Arg8 side-chain away from Glu64 and toward Ile9, and CV *d*_10_ drives salt-bridge breakage by pulling the Glu64 side-chain away from Arg8 and toward Lys65. Using either *d*_9_ or *d*_10_ to break the salt bridge requires the two domains themselves be restrained strongly enough relative to each other. However, this is evidently not the case. Although α_3_ does not break, using these variables was unreliable at breaking the salt bridge; only one successful transition was observed for each of Set 129χ_1_ and 12910χ_1_. Figure S5A,B shows that *d*_6_ does not always break and χ_1_ does not stay in the pocket. In replicas where we see that *d*_6_ does reach its target values, one residue was far away from the other, while the other residue remained extended out in this region creating a blockade that does not allow Phe4 to rotate into the hydrophobic pocket. This causes a problem with *d*_2_ not reaching its target values as well, ultimately leading to a reversion back to the closed state when bias forces are turned off.

Since we found in the second SMD block that the *d*_6_ variable was most effective in breaking the salt bridge, in the next conceptual block of SMD sets in Table 2, we sought to minimize α_3_ deformation by using small values of the harmonic spring constants. Unfortunately none of these sets displayed any successful transitions. Decreasing κ for *d*_6_ from 500 to 50 kcal/mol·nm^2^ still resulted in significant α_3_ deformation, and reducing it further to 5 kcal/mol·nm^2^ did not allow the salt bridge to break at all.

We continue this exploration in the next conceptual block of SMD sets in Table 2 which concerns the biasing schedule. Schedules II, III, and IV, relative to I, devote less time to ramp-up and ramp-down, when the protein conformation is restrained, and relatively more time to steering; that is, the constraints are turned on and off faster but steering is slower. We found that while preserving the hydrogen bonds along the backbone of the α_3_ helix was achieved at times by accelerating the ramp-up and/or slowing down the steering, it causes residues with extended side chains, such as Arg8, Glu64, and Lys60, to position themselves in front of Phe4. This spatial rearrangement obstructs Phe4 from accessing the pocket during the steering process. Although we observed a few successful transitions using these faster-ramp, slower-steer schedules, none were as effective as Schedule I.

We made one attempt to desynchronize the biases of *d*_2_ and *d*_6_, since biasing them simultaneously occasionally broke α_3_. In Set 6–12χ, we first performed a 25 ns ramp up and 25 ns SMD of *d*_6_ from the closed to open characteristic value. We then simultaneously ramped-down *d*_6_ and ramped up *d*_1_, *d*_2_, and χ_1_ for 25 ns, after which *d*_6_ was free and we executed a 25 ns SMD simulation steering *d*_1_, *d*_2_, and χ_1_. Unfortunately the Arg8-Glu64 salt bridge reformed quickly in all cases before χ_1_ could position Phe4, resulting in no successful transitions.

Considering we observed successful transitions by steering all of *d*_1_, *d*_2_, *d*_6_, and χ_1_, and that breakage of the α_3_ helix was observed in many unsuccessful transitions, we then decided to apply a restraint to α_3_ that kept it folded. The variable $L_{\alpha_{3}}$ is defined as the distance between the center of mass of the four N-terminal residues of α_3_ and the center of mass of the four C-terminal residues of α_3_. This variable is not characteristic of the open or closed state (i.e., it has the same value in both states), so it is not one that could be steered. We restrained it to a value of 2.05 nm using a force constant of 500 kJ/mol·nm^2^, which was ramped-up to and ramped-down from together with all other variables’ force constants. This restraint kept α_3_ folded and resulted in successful transitions in SMD simulations steering *d*_1_, *d*_2_, *d*_6_, and χ_1_. We attempted several biasing schedules, as indicated in the last four rows of Table 2.

Figure 6B shows traces of the CVs *d*_1_, *d*_2_, *d*_6_, and χ_1_ when restraining $L_{\alpha_{3}}$ , with ramp-up, steer, and ramp-down phases all 25 ns in duration. At 75 ns, when all restraints are removed, it can be noted that all four CVs remain in their characteristic open-state basins for all five independent simulations. The results suggest that both the initial and final phases of steering need to be carefully managed to avoid unwanted interactions that block key residues, such as Phe4, from reaching their target positions. By increasing the ramp-up time, we were able to achieve a successful conformational transition in all replicas, highlighting the importance of controlling the time scale of steering to balance protein flexibility and structural stability.

**Figure 6:**
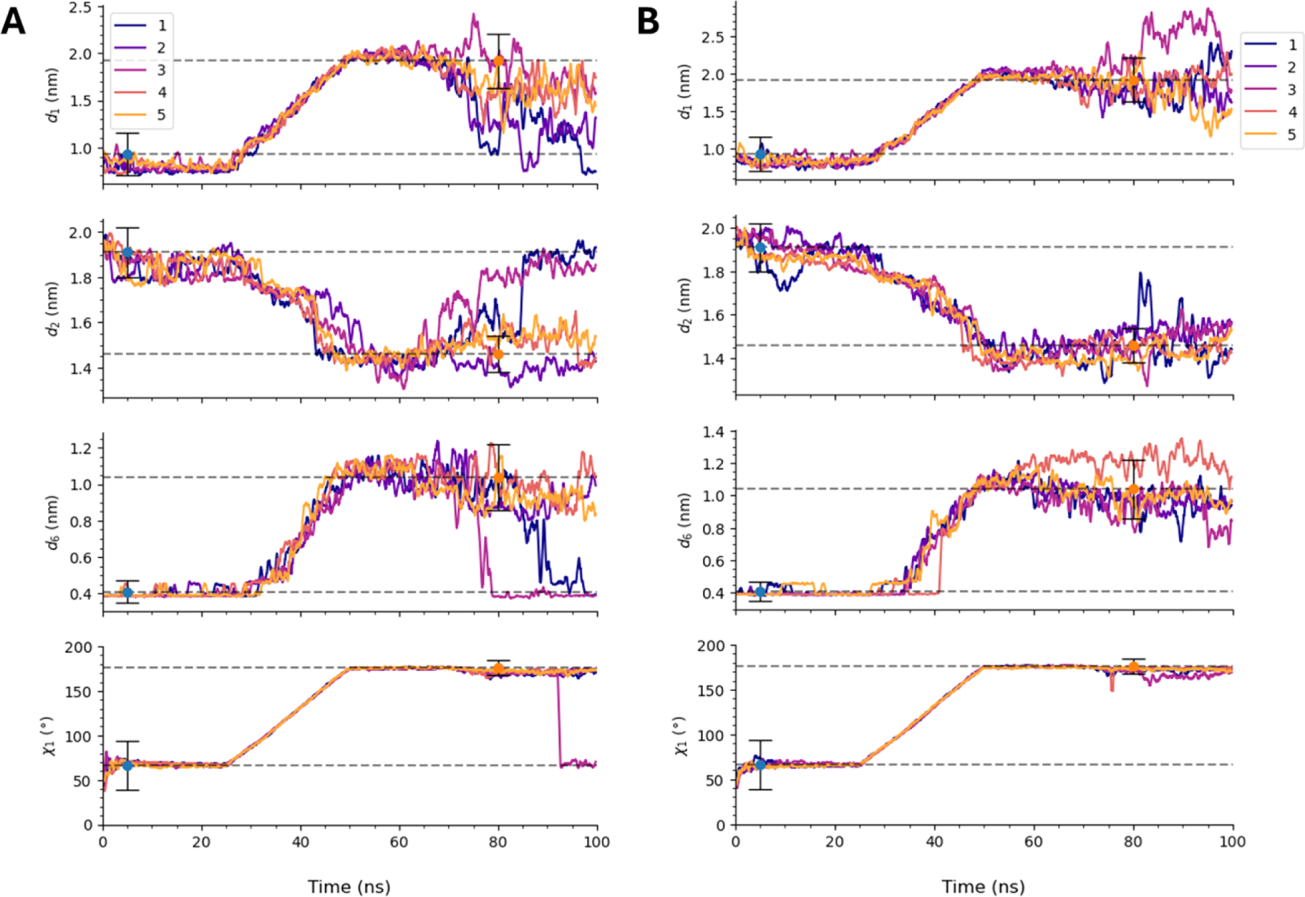
CVs *d*_1_, *d*_2_, *d*_6_, and χ_1_ vs time during SMD simulations where these CVs are steered simultaneously. (A) Set 126χ without the α_3_ helix restraint and (B) set 126α_3_χ with the α_3_ helix restrained. Both sets follow the Schedule I protocol, with ramp-up, steer, and ramp-down phases of 25 ns each. Dashed horizontal lines marked with blue points indicate average values of each CV in the closed state, while lines marked with orange points indicate average values in the open state. Error bars on these indicators represent the standard deviation of each CV in each state. The raw data was smoothed using a rolling function with a window size of 51.

Taken together, these results also illustrate that CVs necessary for faithfully driving the closed-to-open transition of T4L span a hierarchy of length scales. *d*_1_ represents the large-scale relative motion of the two main domains, and *d*_2_ captures the movement of the α_1_ helix relative to α_3_ necessary to place its Phe4 side-chain into the open-state pocket. Access to this pocket is controlled by a salt-bridge between two interdomain residues, and it requires a rotameric transition of the Phe4 side-chain. The simultaneity of the changes in the CVs required for the transition makes its spontaneous observation in MD extremely rare, even though all of them can fluctuate. SMD orchestrates them appropriately to drive the transition, but it must be done carefully to prevent artificial, nonproductive deformations.

Previous work by Ernst et al.^32^ and Post et al.^42^ has demonstrated identifying several important interactions involved in the open-close transition in T4L using Targeted, MD which requires full atomic specification of the target structure and long MD simulations. Here we have considered SMD to bias only a very few low-resolution measurements of the target structure. Our study introduces a distinct strategy that systematically explores and compares multiple steering protocols using a CV set that spans both global and local structural features.

Our strategy for selecting CVs was guided by known features of the hinge-bending motion, experimental smFRET measurements, and structural insights from crystal structures and equilibrium MD. The choice of *d*_1_ and *d*_2_, for instance, was primarily based on capturing the directionality of the large domain motion and selecting variables capable of reproducing this displacement. The selection of smaller-scale CVs such as *d*_6_ and χ_1_ was based on additional observations from simulations and the target structure itself. While *d*_2_ initiates the necessary sliding of α_1_ relative to α_3_, we observed that it does not remain stable throughout the transition and requires a “locking” mechanism to hold it in place. This led us to introduce χ_1_ to enforce the correct rotameric state of Phe4 that locks *d*_2_ in place. These CVs were identified through a combination of equilibrium MD and intermediate SMD trials that revealed when and where additional stabilization was needed.

Thus, while our approach involved systematically testing multiple combinations of CVs, the process was guided by mechanistic insight and structural intuition. In cases where the target structure is only partially known or completely unresolved, one could begin by biasing variables that reflect known large-scale motions and refine the set of CVs by identifying stabilizing interactions between local residues or secondary structure elements, such as the α_1_-α_3_ interface in the case of T4L. The results summarized in Table 1 highlight the importance of using CVs that span multiple scales and also suggest that the number of required SMD trials can be reduced through iterative analysis and refinement based on mechanistic understanding.

#### The Open-to-Closed Transition

Under the conditions in set 126α_3_χ­(d), all simulation replicas successfully transitioned from the closed to the open state. To evaluate the reversibility of the method, we repeated the SMD simulations steering from the open to closed state using both the CV sets that failed (first six sets in Table 2) and the one that successfully induced the transition in the forward direction. We found that sets that failed to induce the closed-to-open transition also failed in the reverse process (Figures S12–S15) where we have 3, 1, 0, 2, 3, and 3 out of 5 successful transitions, respectively. We find that similar to the closed to open transition when we bias *d*_1_, *d*_2_, *d*_6_, and χ_1_ simultaneously in the reverse direction the α_3_ breaks at Phe67 and at times α_1_ becomes deformed during the steering step. In one of the successful steers (replica 5) α_3_ resists breaking at Phe67. The successful closed-to-open set (126α_3_χ­(d)) where we add restraints on $L_{\alpha_{3}}$ , however, achieved 4 out of 5 successful transitions when steering from open to closed (Figure 7). These results reinforce the necessity of incorporating restraints on flexible secondary structures as more strain is being added to the system from increasing the number of CVs being steered.

**Figure 7:**
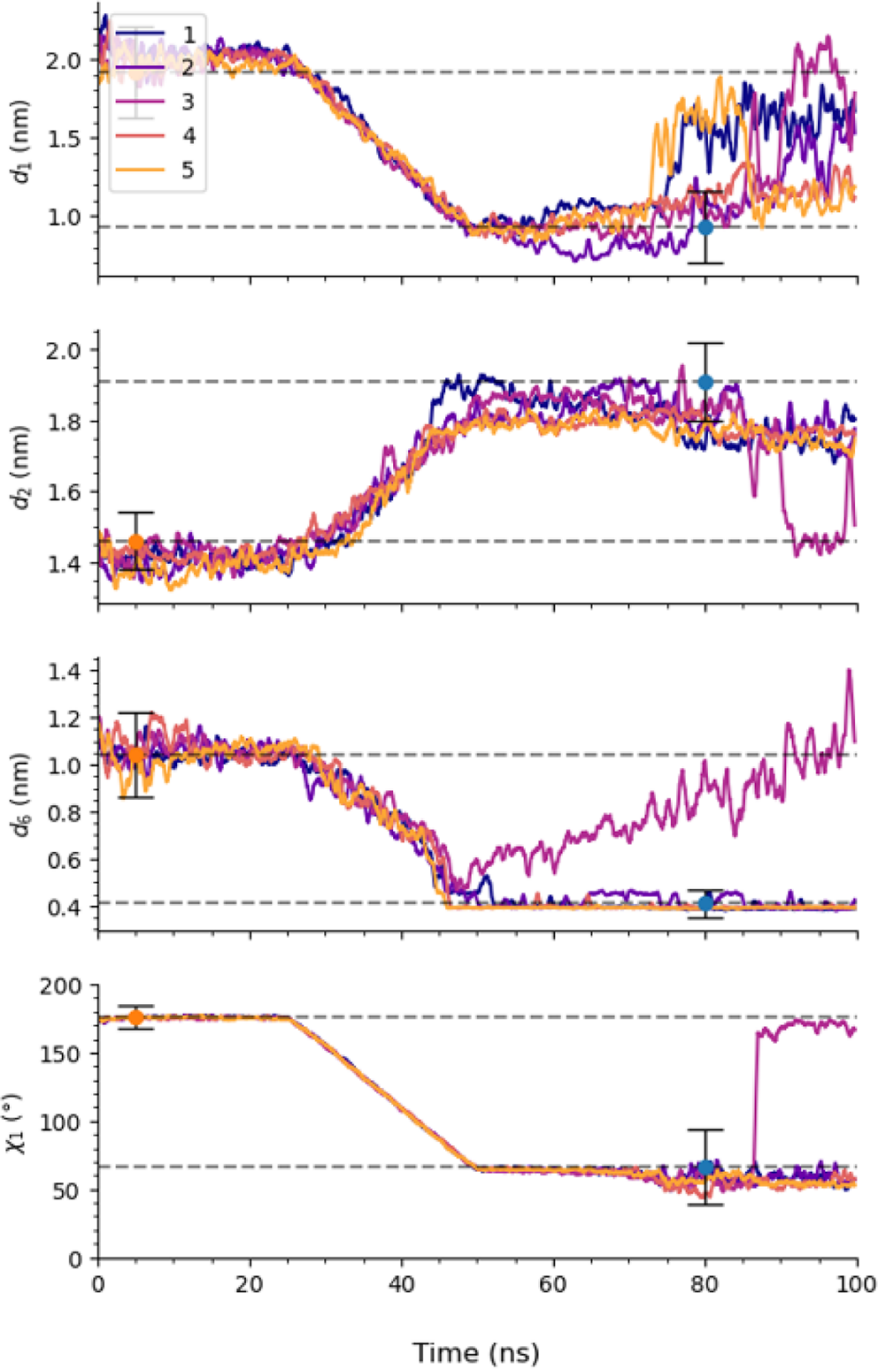
CVs *d*_1_, *d*_2_, *d*_6_, and χ_1_ vs time during SMD simulation set 126α_3_χ­(d), demonstrating the concurrent steering of all four CVs from the wild-type open to closed state of T4L. A force constant of 500 kJ/mol·nm^2^ was applied for distance-based CVs and 1000 kJ/mol·rad^2^ for the torsional angle, with each phase of the steering process-ramp-up, steer, ramp-down, and free-executed in 25 ns intervals. An additional restraint was applied to the α_3_ helix to maintain structural stability by preserving hydrogen bonds along the backbone, using the distance between the center of masses of four residues at the C- and N-termini. The raw data was smoothed using a rolling function with a window size of 51.

To further assess the reliability of the method, we increased the number of replicas for both closed-to-open and open-to-closed transitions from 5 to 10 using the successful CV sets. We maintained a 100% success rate for the closed-to-open transition (Figure S10), while for the reverse process, we achieved 9 out of 10 successful transitions (Figure S16). In the single failed reverse transition, *d*_6_ begins to gradually increase as we ramp down κ from 50 to 75 ns and then remains open causing Phe4 to flip back into the pocket when the bias is completely removed. It is possible that a stiffer spring is needed for steering the *d*_6_ from open to closed since the side-chains were found to fluctuate less in the equilibrium open state than the closed. While achieving perfect reversibility remains challenging, these results suggest that SMD can reliably induce both forward and backward conformational transitions in T4L using low-resolution target information.

#### TAMD Simulations

Thus, far we have identified a minimal set of collective variables that permits us to use SMD to drive conformational transitions in two directions. To assess whether these CVs could induce the same transitions in the absence of any target bias, we performed Temperature-Accelerated Molecular Dynamics (TAMD) simulations. This method leverages an extended Lagrangian formalism to enhance sampling by coupling selected CVs to auxiliary variables at elevated fictitious temperature, allowing the system to explore conformational space more efficiently.^29^

We conducted 15 sets of TAMD simulations where we systematically tuned parameters including the force constant κ, friction γ, relaxation time τ, and fictitious temperature *T*_*f*_, as summarized in Table S1. The time evolution of the CVs and their probability distributions across all parameter sets are presented in Figures S17–S29, and 8. Among the various parameter combinations tested, we found that using γ of 1 ps^–1^ and τ of 100 ps at a *T*_*f*_ of 3000 K, with κ of 500 kJ/mol·nm^2^ for distance variables and 100 kJ/mol·rad^2^ for χ_1_, while restraining Lα_3_ with a harmonic spring of 500 kJ/mol·nm^2^, was optimal to allow all four CVs to simultaneously sample the open state in Replica 1 of Set 15 as depicted in Figure 8.

**Figure 8:**
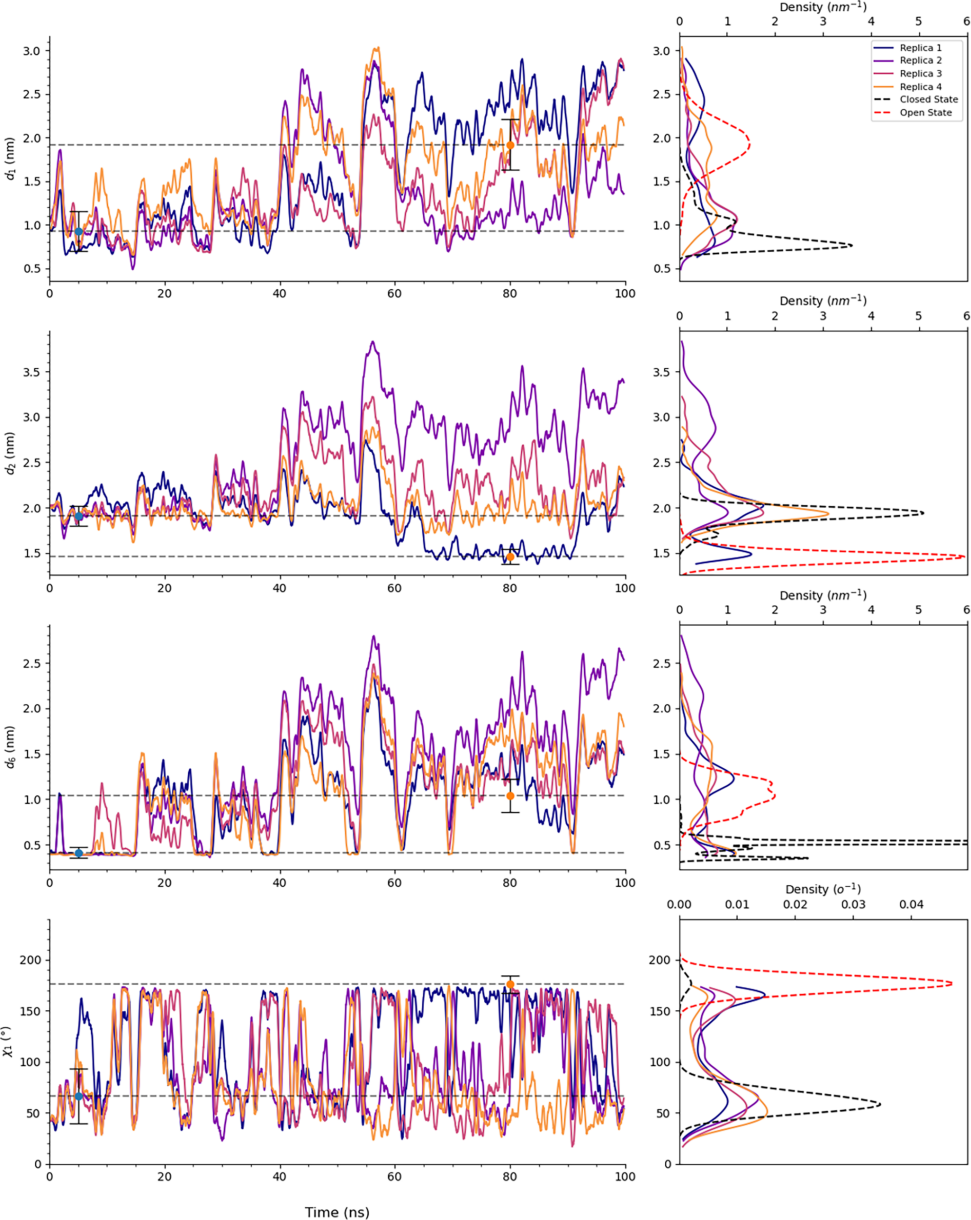
CVs *d*_1_, *d*_2_, *d*_6_, and χ_1_ as a function of time (on the left) from TAMD simulation set 15 (parameters γ = 1 ps^–1^, τ = 100 ps, *T*_*f*_ = 3000 K, κ = 500 kJ/mol·nm^2^ for distance variables and 100 kJ/mol·rad^2^ for χ_1_) and their and corresponding probability density distributions overlaid with equilibrium distribution of the closed and open state (on the right). The raw data to plot the time evolution of the CVs was smoothed using a rolling function with a window size of 51.

To further validate these findings, we extracted ten representative snapshots from the trajectory by filtering frames based on their proximity to open-state equilibrium values of collective variables (*d*_1_, *d*_2_, *d*_6_, and χ_1_), and then ranking them using Euclidean distance from these equilibrium values. These structures then served as initial conditions for ten independent 100 ns equilibrium MD simulations. The resulting probability density distributions for the CVs across all replicas, overlaid with the equilibrium closed- and open-state distributions, are shown in Figure 9. These simulations confirmed that accelerating only these four CVs was sufficient to drive the system into the correct metastable open state. These structures then served as initial conditions for ten independent 100 ns equilibrium MD simulations. The resulting probability density distributions for the CVs across all replicas, overlaid with the equilibrium closed- and open-state distributions, are shown in Figure 9. These simulations confirmed that accelerating only these four CVs was sufficient to drive the system into the correct metastable open state.

**Figure 9:**
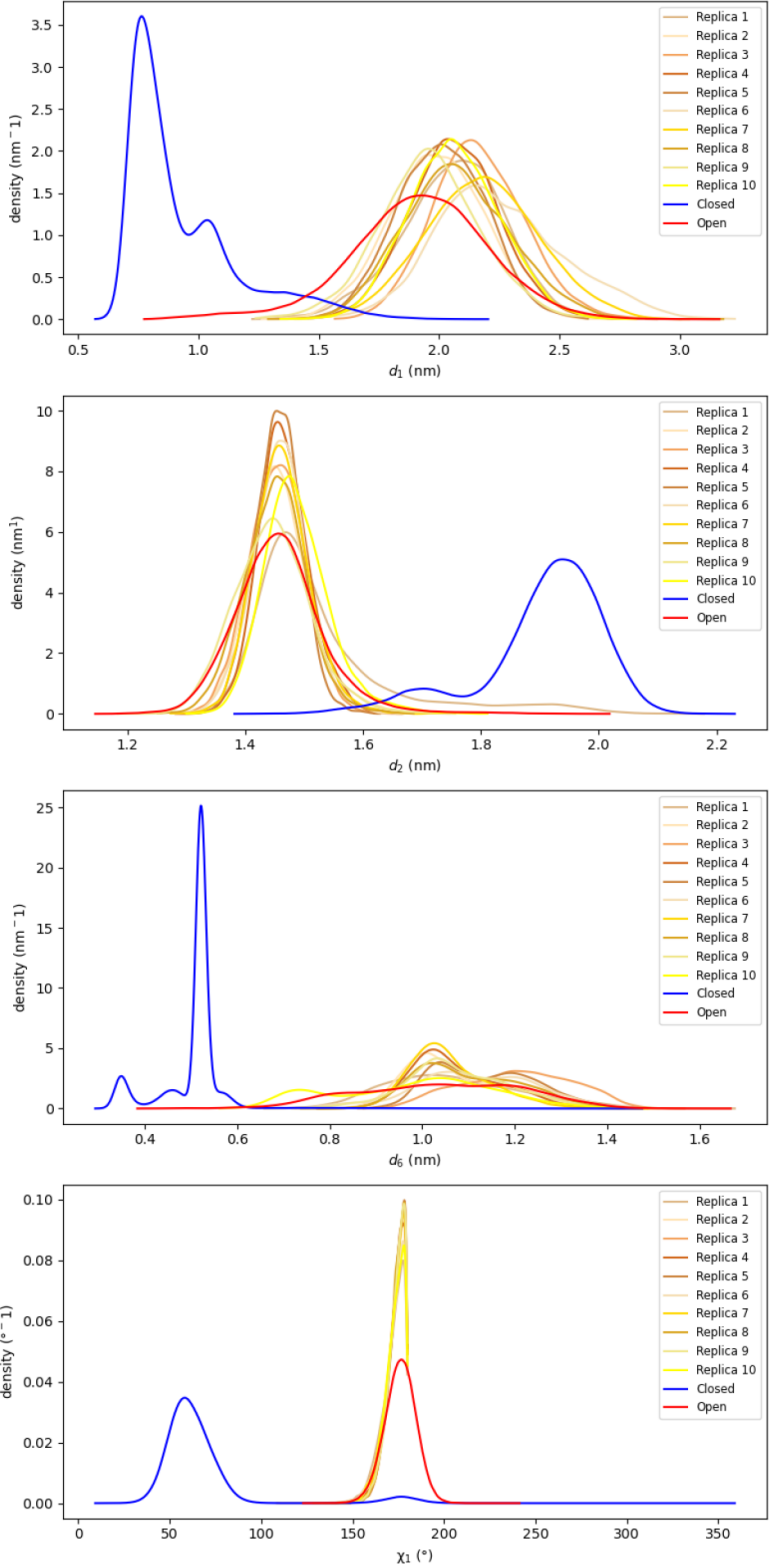
Probability density distributions of interatomic distance variables *d*_1_, *d*_2_, *d*_6_, and torsion angle χ_1_ of Phe4 of T4L from equilibrium MD simulations starting from ten frames extracted from Replica 1 of TAMD simulation set 15.

To assess whether alternative CV sets could achieve similar transitions, we performed additional TAMD simulations using CVs that had failed to induce conformational change in SMD. Specifically, we examined the behavior of *d*_1_ and *d*_2_ (Set 12) and *d*_1_, *d*_2_, and χ_1_ (Set 12χ). The time evolution of *d*_1_, *d*_2_, *d*_6_, and χ_1_ is plotted in Figures S30 and S31. In these cases, TAMD-acceleration of neither *d*_2_ nor *d*_6_ successfully resulted in sampled the open state, and systems instead fluctuated around the closed-state equilibrium. This finding is consistent with our SMD results, which indicated that the motion of *d*_2_ is limited by *d*_6_. Importantly, breaking the Arg8-Glu64 salt bridge appears to be a prerequisite for conformational change. Accelerating large-scale domain motions of *d*_1_ and *d*_2_ alone, without first disrupting this electrostatic interaction, was insufficient to drive the transition.

## Conclusions

Understanding conformational transitions between metastable states requires carefully chosen collective variables (CVs) and carefully tuned steering parameters in enhanced sampling methods. In this study, we identified four essential interatomic distance CVs and one side-chain rotameric angle that reliable allow bidirectional conformational changes in T4 lysozyme in all-atom steered MD (SMD) simulations. A key finding is the observation that large-scale domain motions alone are insufficient without steering local side-chain rearrangements. Key stabilizing interactions, such as the Arg8-Glu64 salt bridge and the rotation of Phe4, regulate the transition, while careful tuning of SMD parameters and flexible helix restraints prevent structural distortions. Temperature-Accelerated MD (TAMD) further validated these CVs, confirming their role in facilitating the transition without explicit target bias and highlighting solvent contributions. By integrating SMD and TAMD, our study provides a framework for systematically identifying mechanistic determinants of protein conformational transitions, applicable to other dynamic biomolecular systems.

## Supplementary Material



## Notes

Other figures of time evolution of CVs during SMD sets of closed-to-open and open-to-closed transitions, as well as a table of TAMD parameter benchmark figures for each TAMD simulation set (PDF)
