# Supplementary material for: Minimal Collective Variables for Conformational Transitions in Steered and Temperature-Accelerated MD Simulations: A T4 Lysozyme Case Study
Source: J Phys Chem B. 2025 May 16;129(21):5176–88. doi: 10.1021/acs.jpcb.5c01129 (PMC12128028; doi:10.1021/acs.jpcb.5c01129)
Supplement: Supplementary file 1 [file jp5c01129_si_001.pdf]

Supporting Information: Minimal Collective Variables for Conformational Transitions in Steered and Temperature-Accelerated MD Simulations: A T4 Lysozyme Case Study

Salsabil Abou-Hatab and Cameron F. Abrams*

Department of Chemical and Biological Engineering, Drexel University, Philadelphia, Pennsylvania

E-mail: cfa22@drexel.edu

SMD simulations of the Closed to Open Transition

Traces of CV distances and the χ_1 angle of Phe4 vs simulation time for the steered MD (SMD) simulation sets 12, 12χ, 126, 186, 129χ, 129-10χ, 126χ-(F1), 126χ-(F2), 126χ-(S1), 126χ-(S2), 126χ-(S3), 6-12χ, 126$\alpha_3\chi$-(a), 126$\alpha_3\chi$-(b), and 126$\alpha_3\chi$-(c), steering T4L from its closed to open state, are plotted in Figs. S1 to S9. Results are discussed in the main paper.

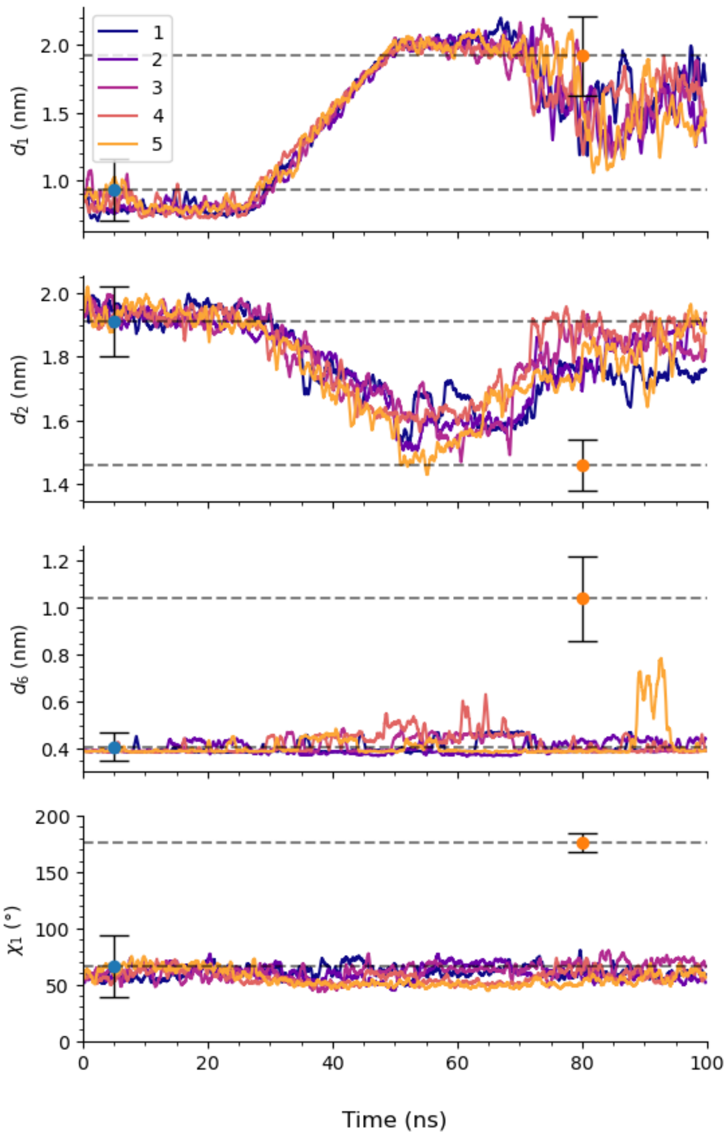

Figure S1: CVs d_1, d_2, d_6, and χ_1 v. time during SMD simulation set 12, where d_1 and d_2 were simultaneously biased to transition from the wild-type open to closed state of T4L. A force constant of 500 kJ/mol·nm^2 was applied to distance-based CVs and 1000 kJ/mol·rad^2 to the torsional angle, with each steering phase—ramp-up, steer, ramp-down, and free—executed in 25 ns increments.

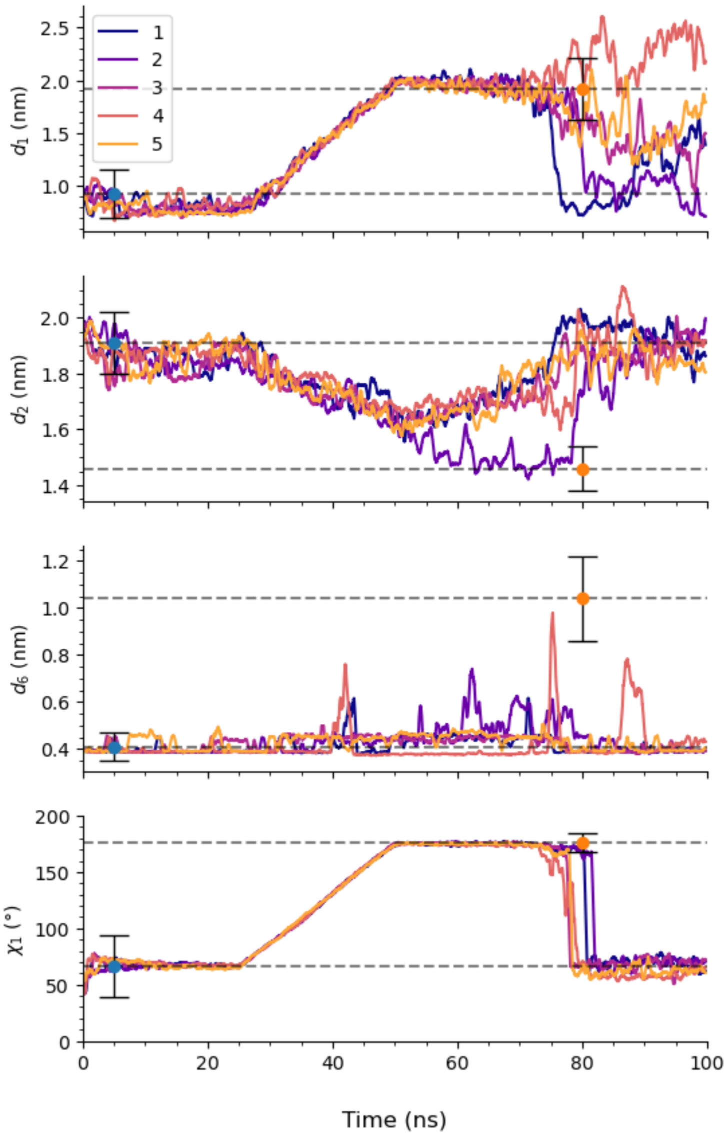

Figure S2: CVs d_1, d_2, d_6, and χ_1 v. time during SMD simulation set 123, where d_1, d_2, and χ_1 were simultaneously biased to transition from the wild-type open to closed state of T4L. A force constant of 500 kJ/mol·nm^2 was applied to distance-based CVs and 1000 kJ/mol·rad^2 to the torsional angle, with each steering phase—ramp-up, steer, ramp-down, and free—executed in 25 ns increments.

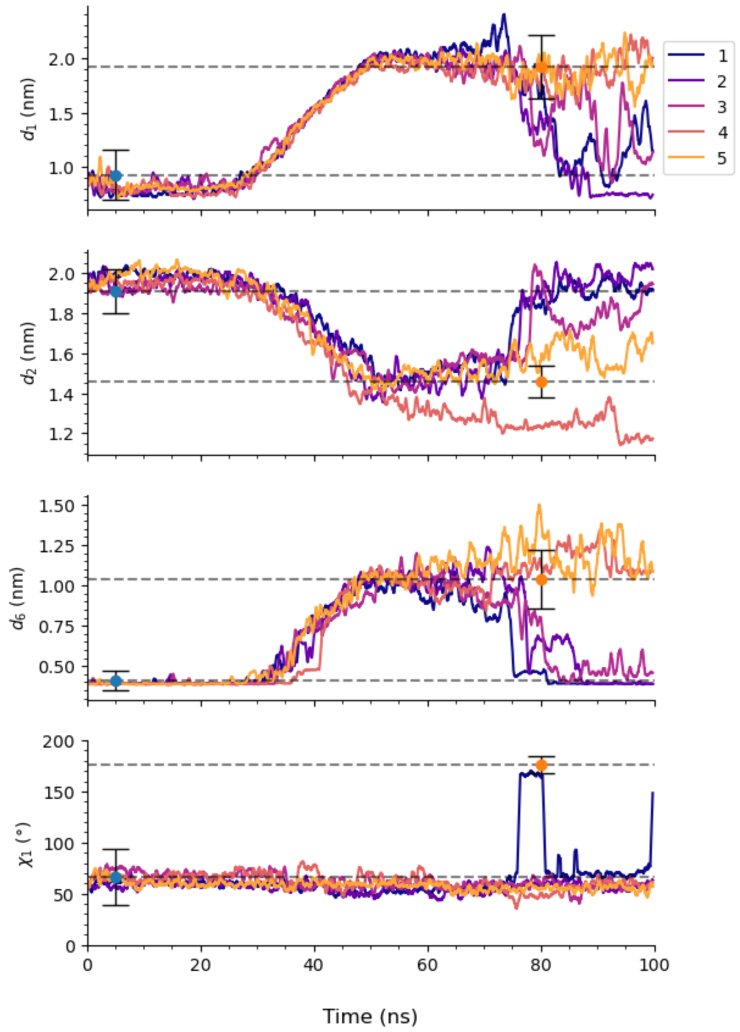

Figure S3: CVs d_1, d_2, d_6, and χ_1 v. time during SMD simulation set 126, where d_1, d_2, and d_6 were simultaneously biased to transition from the wild-type open to closed state of T4L. A force constant of 500 kJ/mol·nm^2 was applied to distance-based CVs, with each steering phase—ramp-up, steer, ramp-down, and free—executed in 25 ns increments.

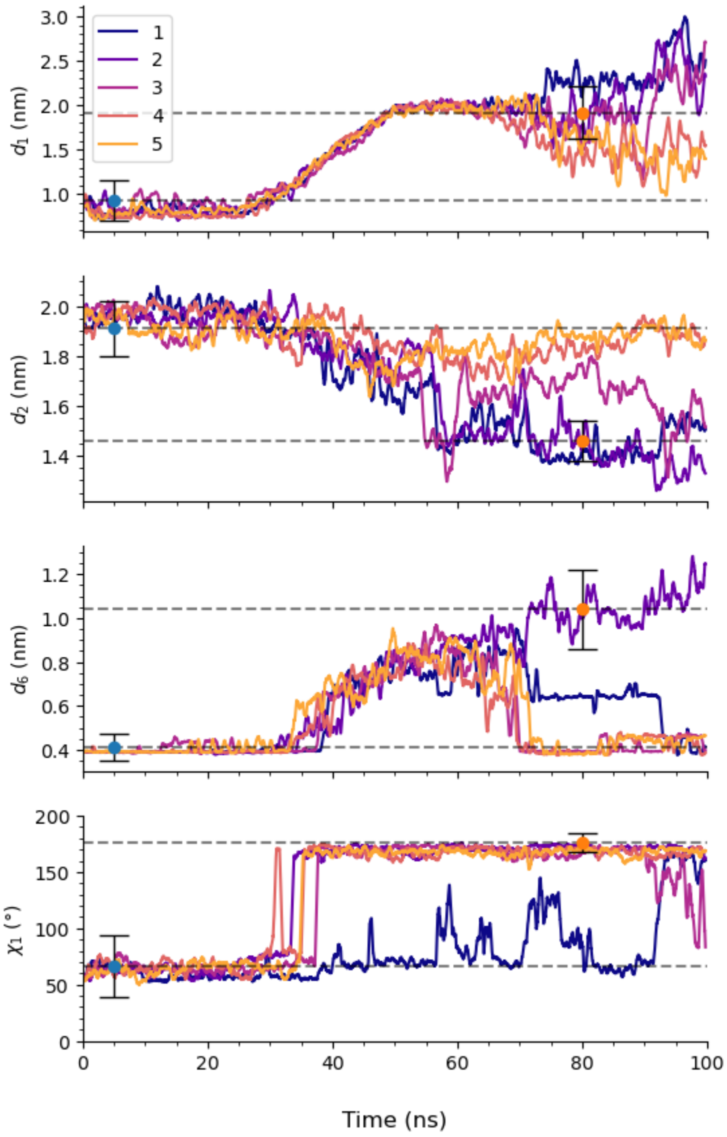

Figure S4: CVs d_1, d_2, d_6, and χ_1 v. time during SMD simulation set 168, where d_1, d_6, and d_8 were simultaneously biased to transition from the wild-type open to closed state of T4L. A force constant of 500 kJ/mol·nm^2 was applied to distance-based CVs and 1000 kJ/mol·rad^2 to the torsional angle, with each steering phase—ramp-up, steer, ramp-down, and free—executed in 25 ns increments.

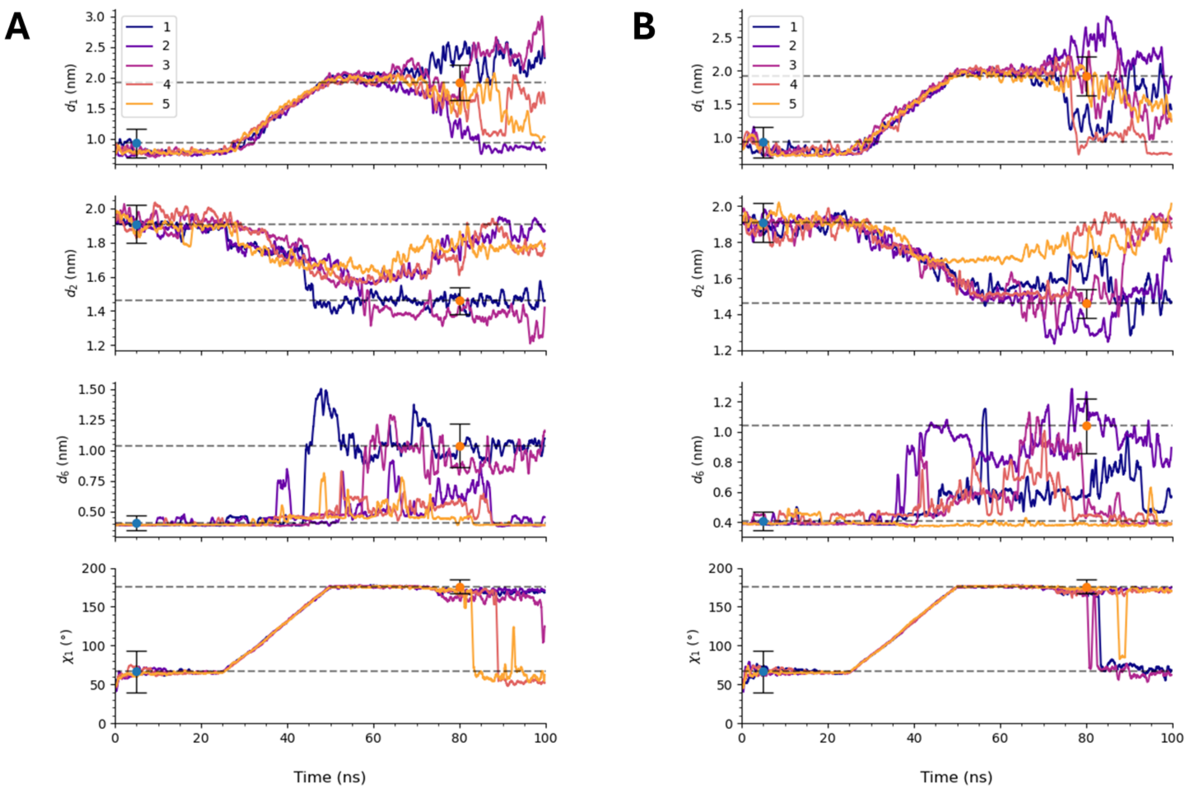

Figure S5: CVs d_1, d_2, d_6, and χ_1 vs. time in SMD simulation sets (A) 129χ and (B) 129-10-χ, where d_9 is biased in the first set and both d_9 and d_{10} are biased simultaneously in the latter to disrupt the salt bridge while steering d_1 and d_2 to transition from the wild-type open to closed state of T4L. A force constant of 500 kJ/mol·nm^2 was applied to distance-based CVs and 1000 kJ/mol·rad^2 to the torsional angle, with each steering phase—ramp-up, steer, ramp-down, and free—executed in 25 ns increments.

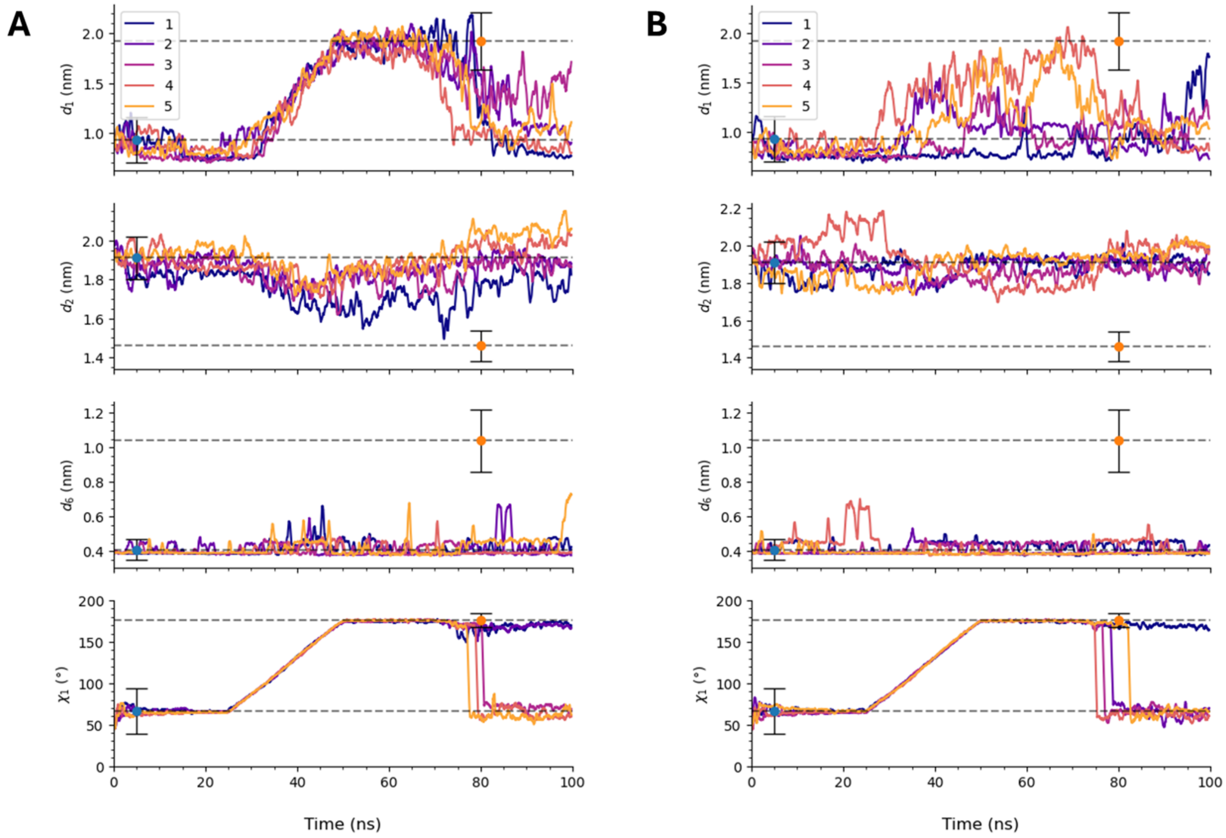

Figure S6: CVs d_1, d_2, d_6, and χ_1 vs. time in SMD simulation sets (A) F1 and (B) F2, benchmarking the effects of force strength. Force constants in F1 were set to 20, 10, and 5 kJ/mol·nm^2 for d_1, d_2, and d_6, respectively, and to 200, 100, and 50 kJ/mol·nm^2 in F2, with a constant of 1000 kJ/mol·rad^2 for χ_1 in both sets. Each steering phase—ramp-up, steer, ramp-down, and free—was executed in 25 ns intervals.

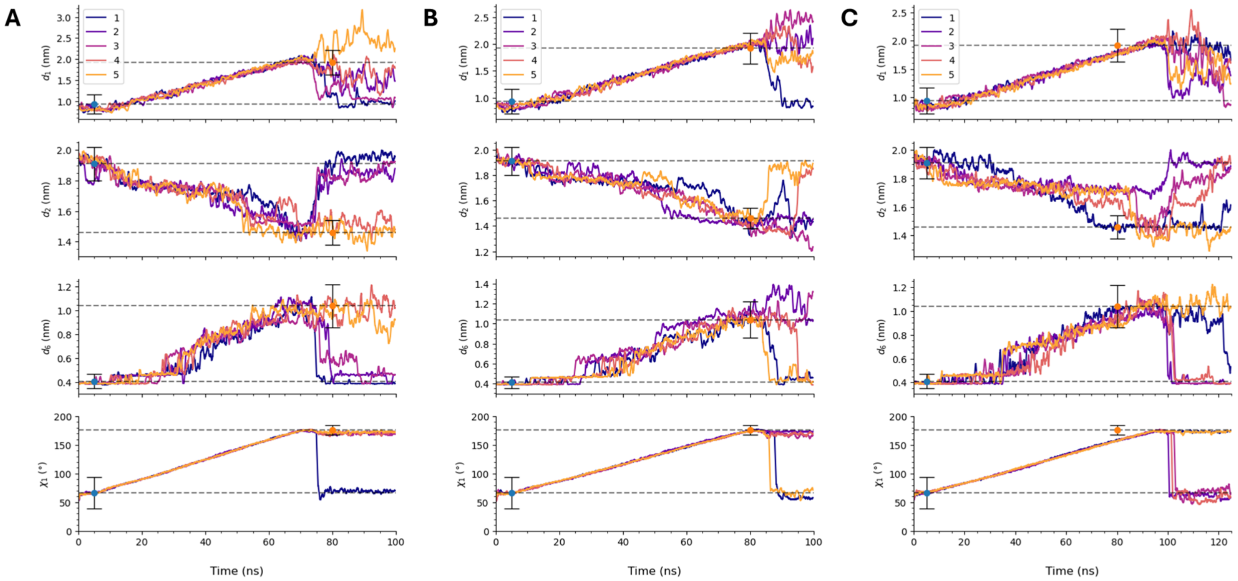

Figure S7: CVs d_1, d_2, d_6, and χ_1 vs. time in SMD simulation sets 126χ (A–C), where each set uses a modified steering protocol to gradually reduce the pulling speed by extending the steering duration. In these simulations, the SMD protocol ramps up the force constant (κ) over 5 ns, simultaneously steers the CVs for (A) 65 ns, (B) 75 ns, and (C) 90 ns, and then ramps down κ over another 5 ns. The system is left to equilibrate freely for 25 ns, 15 ns, and 25 ns, respectively.

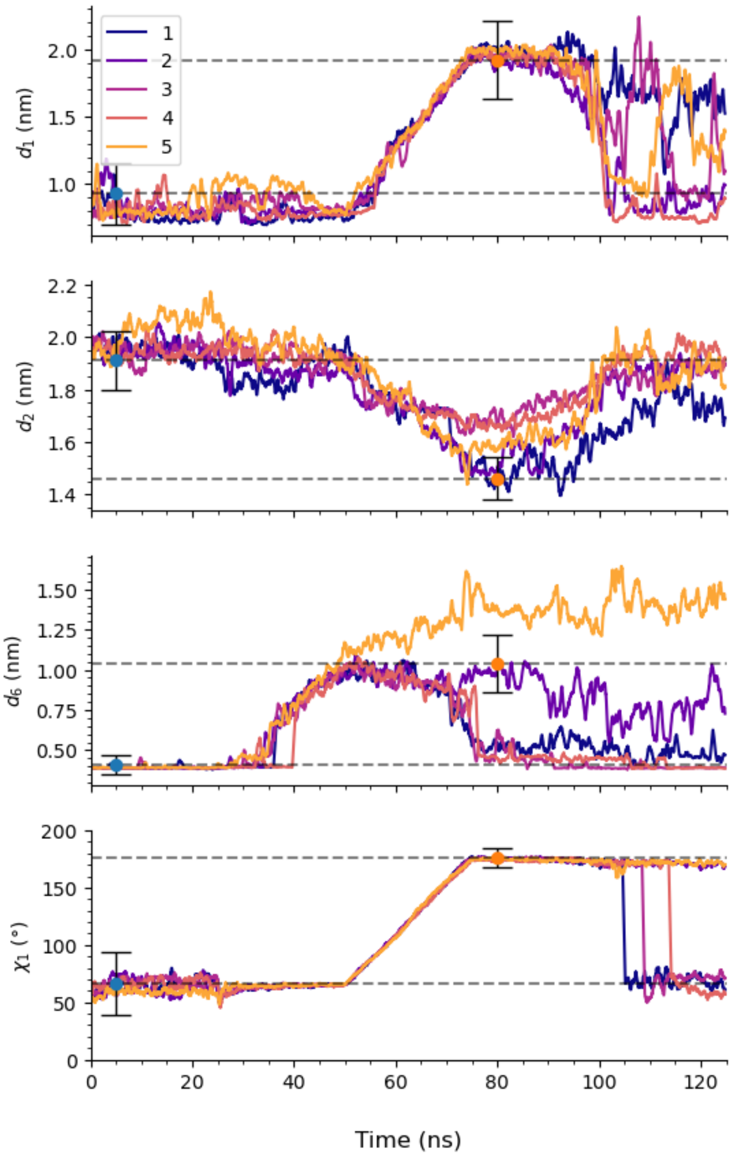

Figure S8: CVs d_1, d_2, d_6, and χ_1 v. time in SMD simulation set 126χ, illustrating the coordinated steering of all four CVs from the wild-type closed to open state of T4L. A force constant of 500 kJ/mol·nm^2 was applied for distance-based CVs and 1000 kJ/mol·rad^2 for the torsional angle, with each phase (ramp-up, steer, ramp-down, and free) carried out in 25 ns intervals. A stepwise steering protocol was used, where κ was ramped up over 25 ns, followed by steering d_6 for 25 ns to break the salt bridge. After d_6 reached the target value, the bias was gradually reduced over 25 ns while d_1, d_2, and χ_1 were simultaneously steered from 50 to 75 ns. Finally, the bias was ramped down over 25 ns, and all four CVs were sampled without bias in the last 25 ns

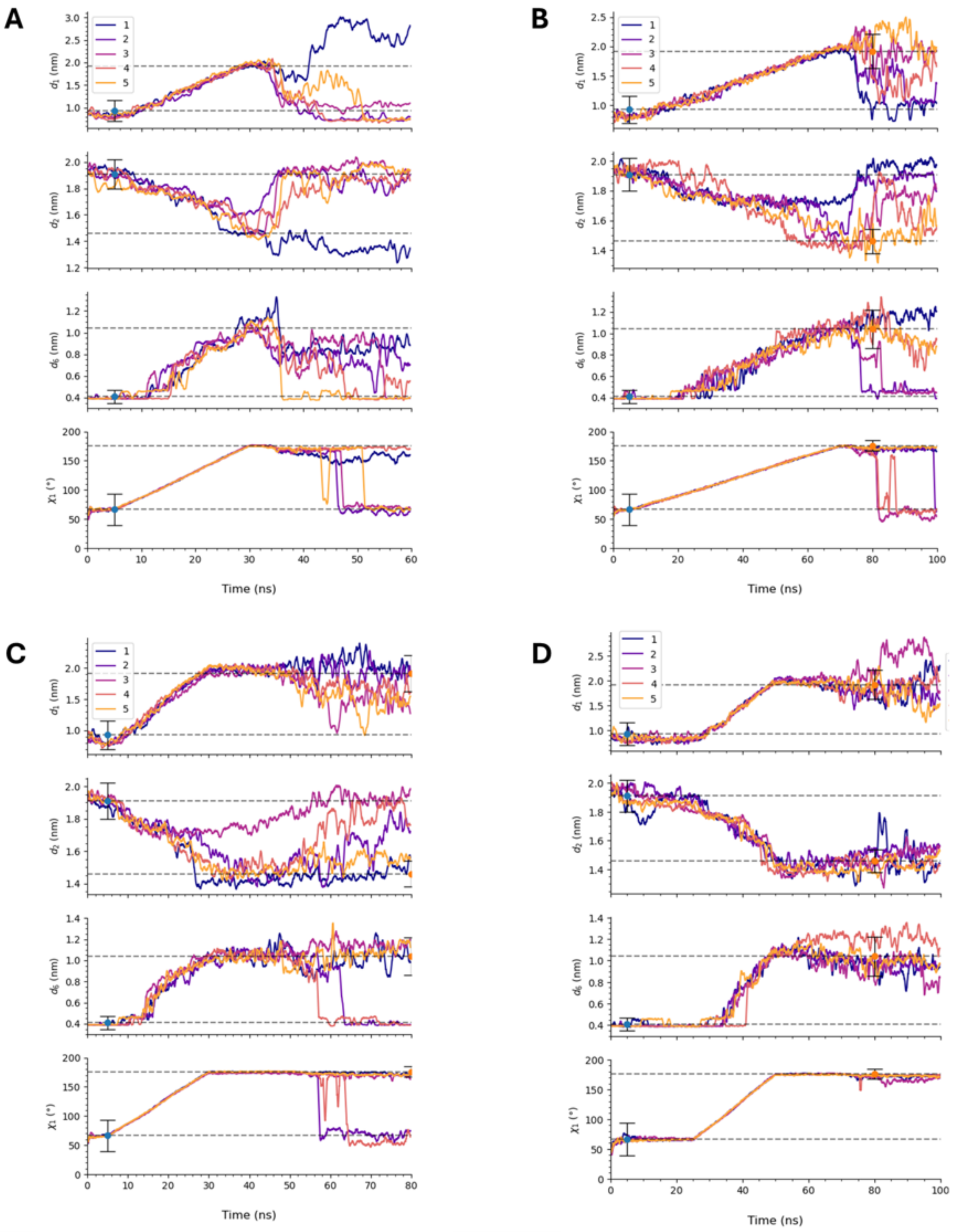

Figure S9: CVs d_1, d_2, d_6, and χ_1 vs. time in SMD simulation sets $126\alpha_3\chi$ (A–D), where all four variables are steered with an additional restraint on the α_3 helix to maintain hydrogen bonds along its backbone by controlling the end-to-end center of mass distance. We evaluate the effects of varying steering durations with different timings for the ramp-up, steer, ramp-down, and free phases: (A) 5-25-5-25 ns, (B) 5-65-5-25 ns, (C) 5-25-25-25 ns, and (D) 25-25-25-25 ns, respectively, in achieving a successful conformational transition between the two metastable states of T4L.

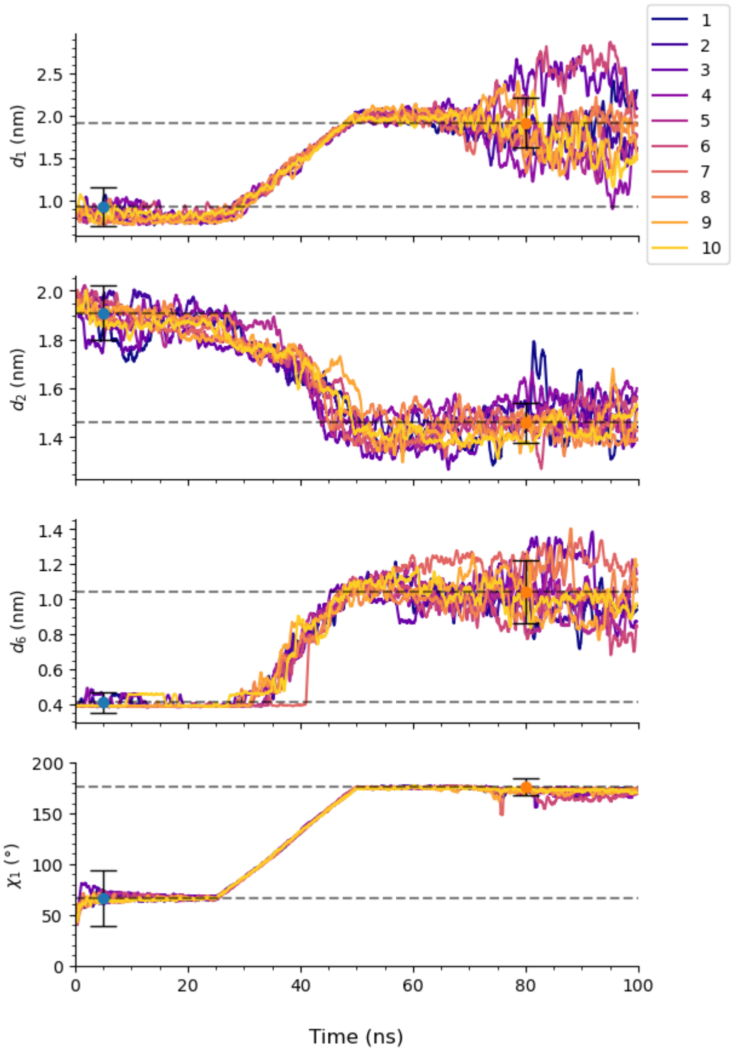

Figure S10: CVs d_1, d_2, d_6, and χ_1 v. time during SMD simulation set $126\alpha_3\chi$(d), demonstrating the concurrent steering of all four CVs from the wild-type closed to open state of T4L. A force constant of 500 kJ/mol·nm^2 was applied for distance-based CVs and 1000 kJ/mol·rad^2 for the torsional angle, with each phase of the steering process—ramp-up, steer, ramp-down, and free—executed in 25 ns intervals. An additional restraint was applied to the α_3 helix to maintain structural stability by preserving hydrogen bonds along the backbone, using the distance between the center of masses of four residues at the C- and N-termini. The raw data was smoothed using a rolling function with a window size of 51. Number of replicas were increased from 5 to 10.

SMD simulations of the Open to Closed Transition

Traces of distance CV's and χ_1 of Phe4 vs time for SMD simulation sets 1, 2, 12, 12χ, 126, and 126χ, for steering T4L from its open to closed state, are shown in Figs. S11 to S15. Results are discussed in the main paper.

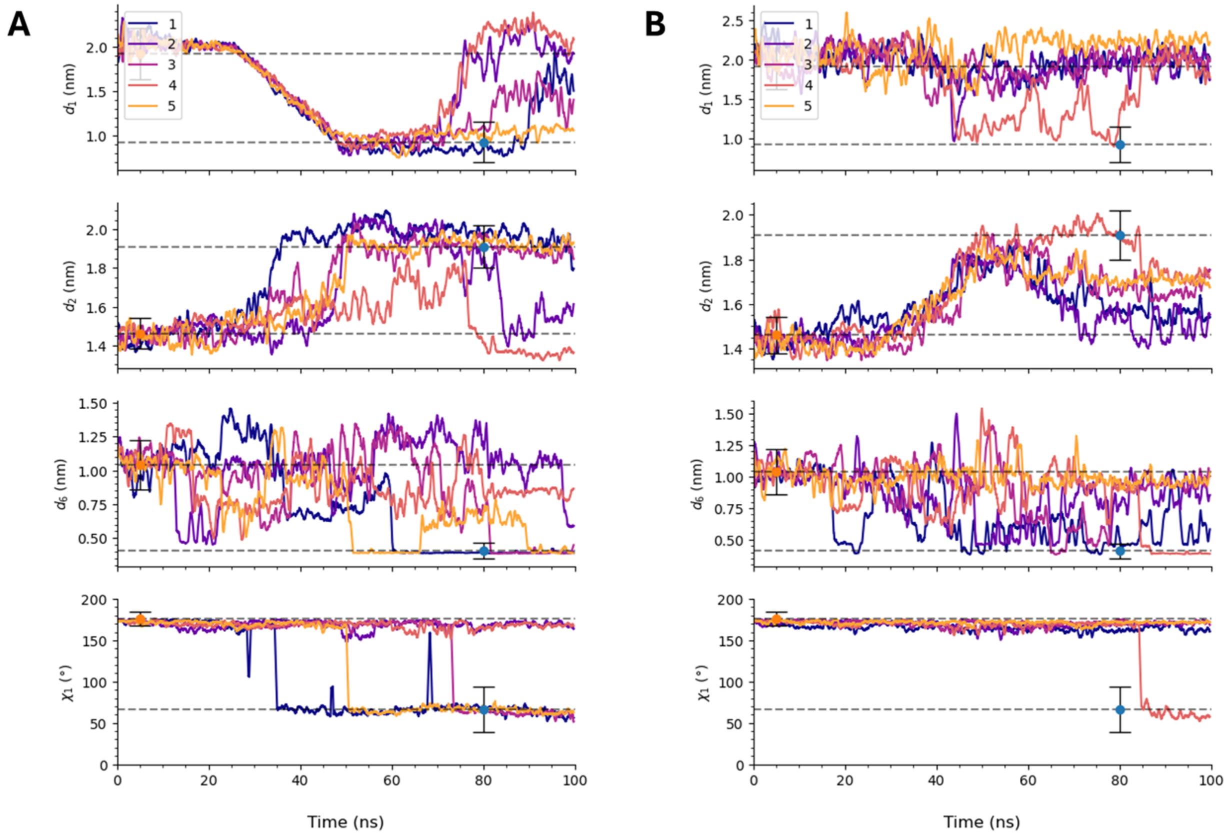

Figure S11: CVs d_1, d_2, d_6, and χ_1 vs. time during SMD simulation sets 1 and 2, illustrating the behavior of each variable over time as a single CV—either (A) d_1 or (B) d_2—is steered independently from the wild-type open to closed state of T4L using a force constant of 500 kJ/mol·nm^2. The four stages of steered MD—ramp-up, steer, ramp-down, and free—were executed in 25 ns intervals.

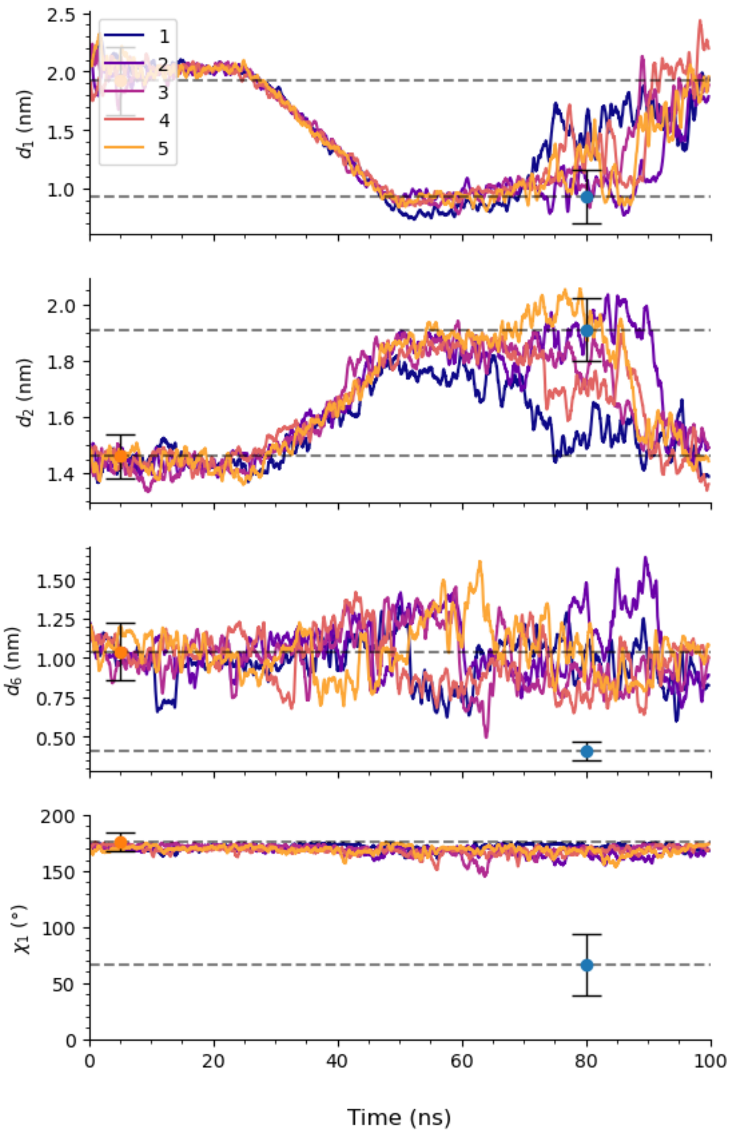

Figure S12: CVs d_1, d_2, d_6, and χ_1 v. time during SMD simulation set 12, where d_1 and d_2 were simultaneously biased to transition from the wild-type open to closed state of T4L. A force constant of 500 kJ/mol·nm^2 was applied to distance-based CVs and 1000 kJ/mol·rad^2 to the torsional angle, with each steering phase—ramp-up, steer, ramp-down, and free—executed in 25 ns increments.

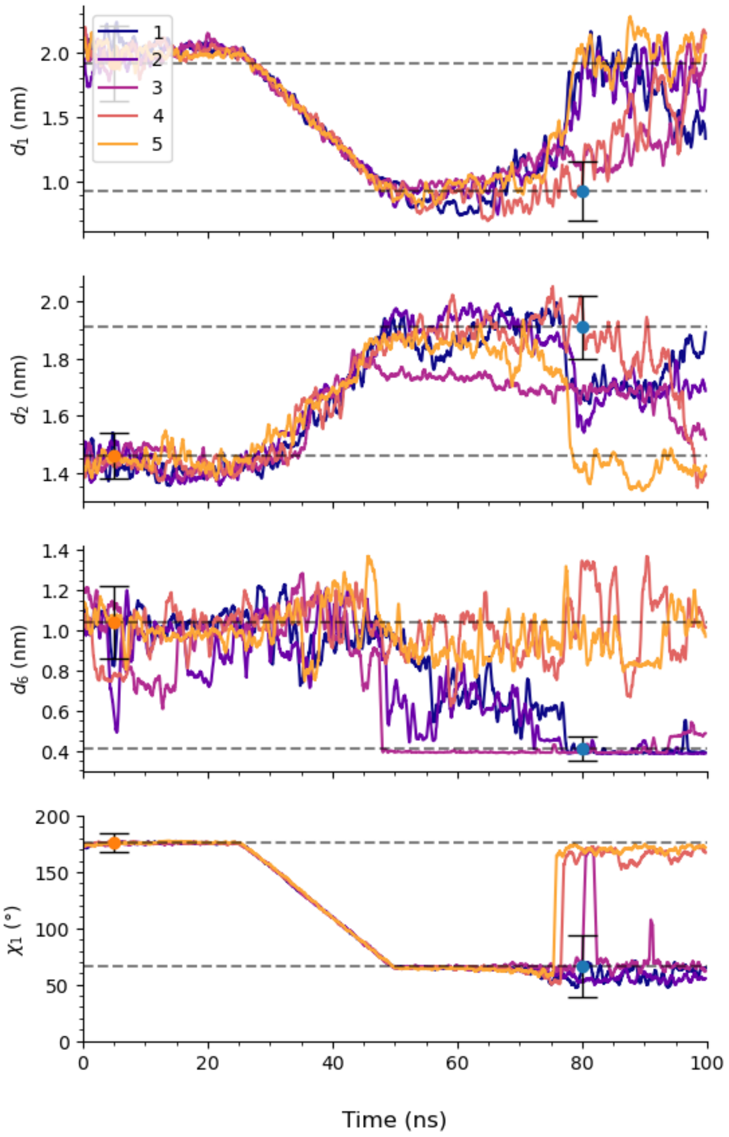

Figure S13: CVs d_1, d_2, d_6, and χ_1 v. time during SMD simulation set 123, where d_1, d_2, and χ_1 were simultaneously biased to transition from the wild-type open to closed state of T4L. A force constant of 500 kJ/mol·nm^2 was applied to distance-based CVs and 1000 kJ/mol·rad^2 to the torsional angle, with each steering phase—ramp-up, steer, ramp-down, and free—executed in 25 ns increments

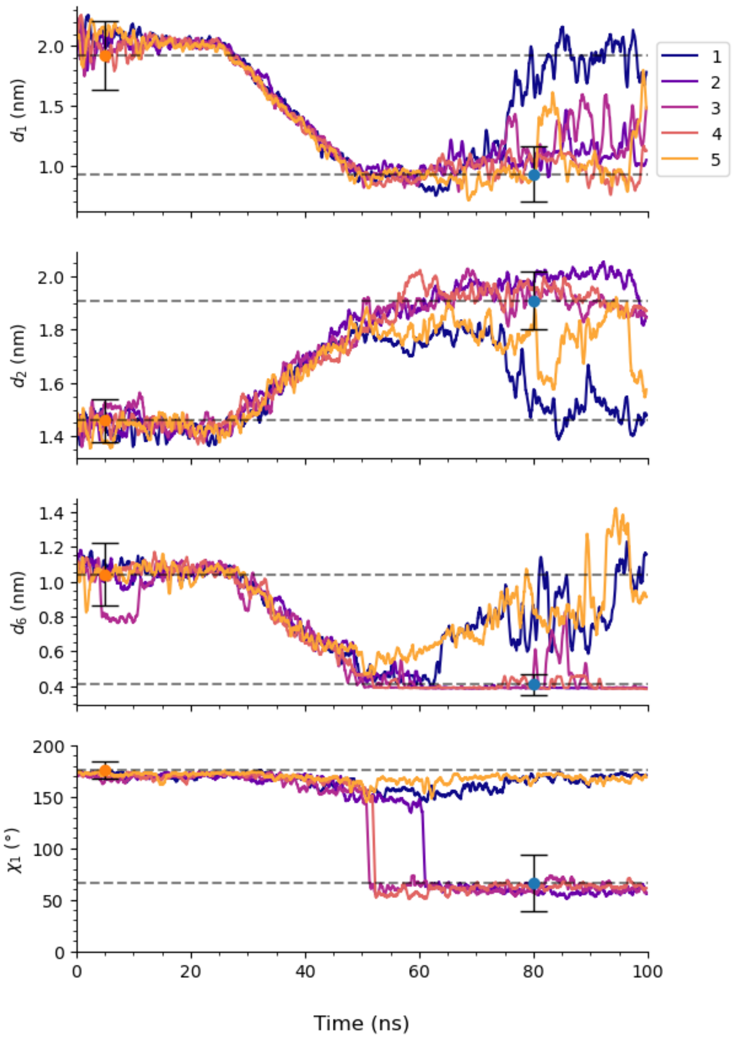

Figure S14: CVs d_1, d_2, d_6, and χ_1 v. time during SMD simulation set 126, where d_1, d_2, and d_6 were simultaneously biased to transition from the wild-type open to closed state of T4L. A force constant of 500 kJ/mol·nm^2 was applied to distance-based CVs and 1000 kJ/mol·rad^2 to the torsional angle, with each steering phase—ramp-up, steer, ramp-down, and free—executed in 25 ns increments

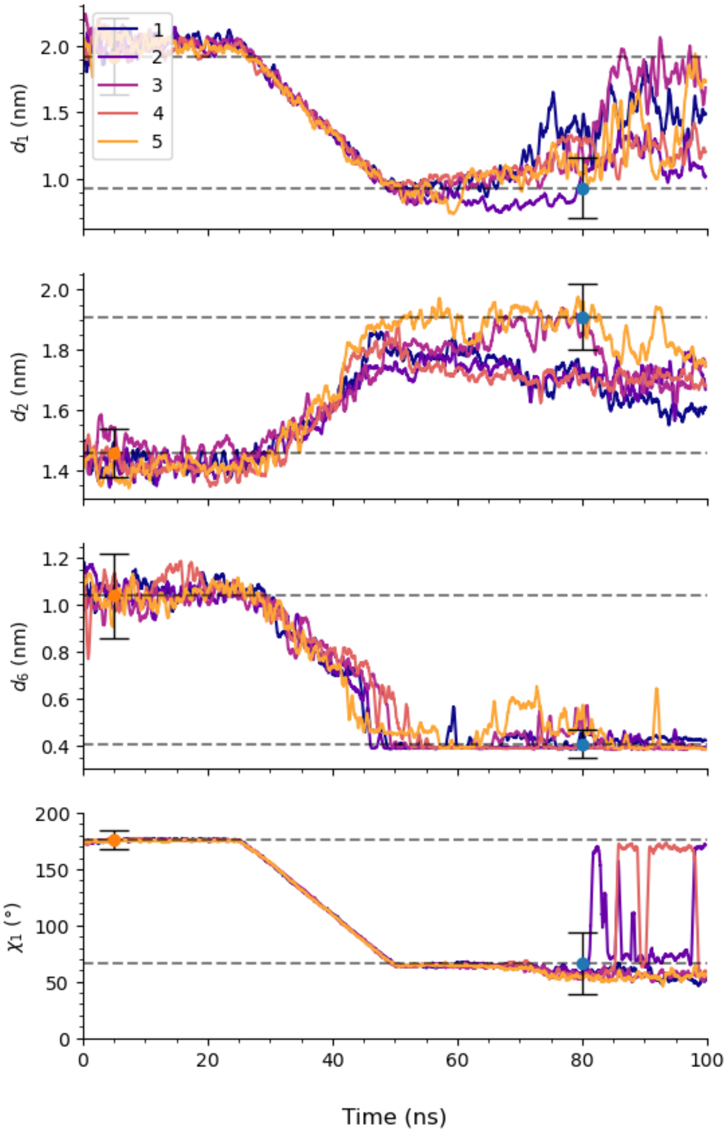

Figure S15: CVs d_1, d_2, d_6, and χ_1 v. time throughout SMD simulation set 126χ, demonstrating the concurrent steering of all four CVs from the wild-type open to closed state of T4L. A force constant of 500 kJ/mol·nm^2 was applied for distance-based CVs and 1000 kJ/mol·rad^2 for the torsional angle, with each phase of the steering process—ramp-up, steer, ramp-down, and free—executed in 25 ns intervals.

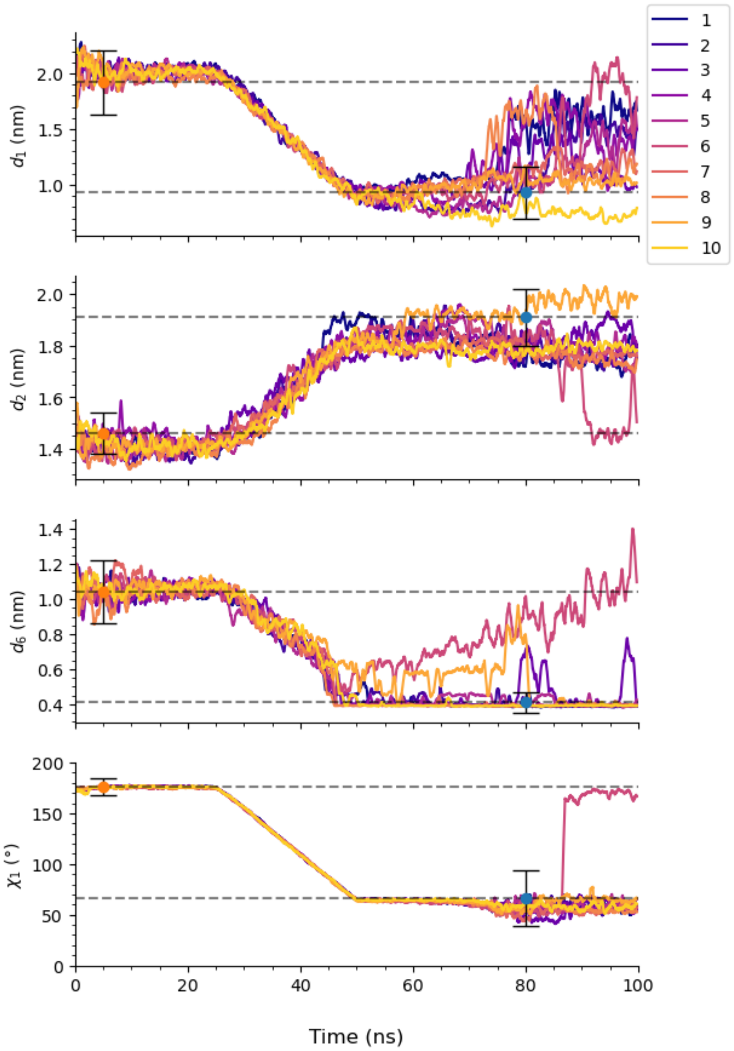

Figure S16: CVs d_1, d_2, d_6, and χ_1 v. time during SMD simulation set $126\alpha_3\chi$(d), demonstrating the concurrent steering of all four CVs from the wild-type open to closed state of T4L. A force constant of 500 kJ/mol·nm^2 was applied for distance-based CVs and 1000 kJ/mol·rad^2 for the torsional angle, with each phase of the steering process—ramp-up, steer, ramp-down, and free—executed in 25 ns intervals. An additional restraint was applied to the α_3 helix to maintain structural stability by preserving hydrogen bonds along the backbone, using the distance between the center of masses of four residues at the C- and N-termini. The raw data was smoothed using a rolling function with a window size of 51. The number of replicas was increased from 5 to 10.

TAMD simulations

Tuning of parameters for the Temperature-Accelerated MD (TAMD) simulations was peformed over fourteen sets of TAMD simulations by varying the force constant, κ, friction, γ, relaxation time, τ, and fictitious temperature, T_f, as tabulated in Table 1.

Table 1: Tuning of Temperature Accelerated MD simulation parameters: force constant, κ, fictitious temperature, T_f, fictitious friction, γ, and relaxation time, τ. κ is reported in units of kJ/mol·nm^2 for distance variables and kJ/mol·rad^2 for torsion angles (e.g., χ_1). Biasing CVs d_1, d_2, d_6, and χ_1 with harmonic restrain on L_{a_3}

**Set**	**CVs and κ**	T_f **(K)**	γ **(ps^{-1})**	τ **(ps)**
1–4	d_1, d_2, d_6: 500; χ_1: 1000	all: 3000	1	1, 10, 100, 1000
5–8	d_1, d_2, d_6: 500; χ_1: 1000	all: 3000	10	0.1, 1, 10, 100
9	d_1, d_2, d_6: 500; χ_1: 1000	all: 3000	0.1	100
10	d_1, d_2, d_6: 500; χ_1: 1000	all: 6000	1	100
11	d_1, d_2, d_6: 500; χ_1: 1000	d_1, χ_1: 3000; d_2, d_6:6000	1	100
12	d_1, d_2, d_6: 500; χ_1: 1000	d_1, d_2: 3000; d_6, χ_1: 6000	1	100
13	d_1, d_2, d_6, χ_1: 1000	all: 3000	1	100
14	d_1, d_2, d_6: 50; χ_1: 100	all: 3000	1	100
15	d_1, d_2, d_6: 500; χ_1: 100	all: 3000	1	100

In sets 1 to 4 (Figs. S17–S20), we accelerate the four CVs at $T = 3000$ K with a $\gamma = 1$ ps^{-1} while increasing τ by a factor of 10 from 1 to 1000 ps respectively. We found that at $\tau = 1$ ps, all replicas remain in the closed state, except for one case where d_1 fluctuates to open-state values, while χ_1 explores larger torsion angles within the closed state region. Increasing τ to 10 ps enhances thermal fluctuations but remains insufficient for significant sampling of the open state. At $\tau = 100$ ps, distance variables reach the open-state equilibrium, but χ_1 does not, and d_2 still fails to frequently attain its target values. Moreover, all four variables do not sample the open state simultaneously. When τ is further increased to 1000 ps, motion is suppressed, keeping all variables in the closed-state equilibrium. We found that $\tau = 100$ ps allows all four CVs to sample the open-state equilibrium values, albeit not concurrently, suggesting the need for further optimization of TAMD parameters.

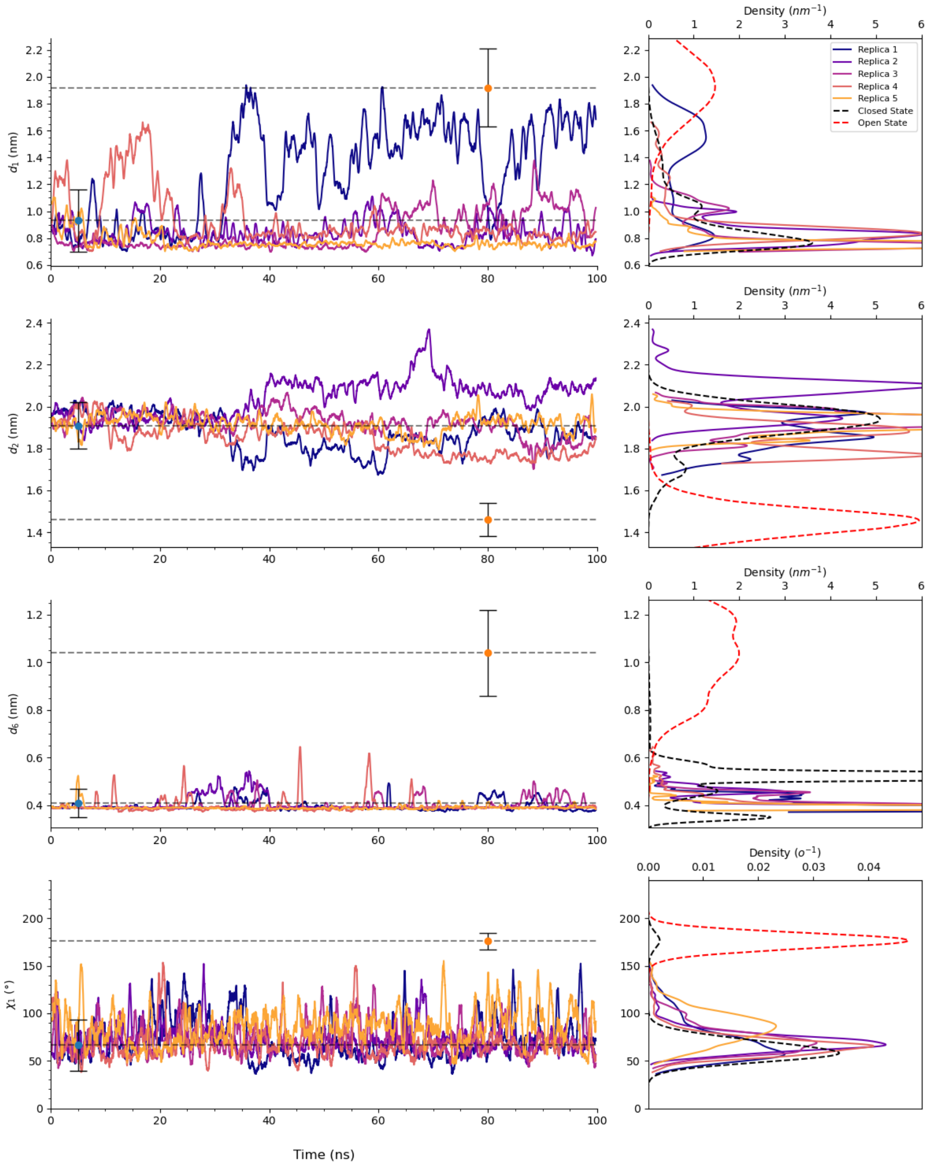

Figure S17: CVs d_1, d_2, d_6, and χ_1 v. time (on the left) from TAMD simulation set 1 (parameters $\gamma = 1$ ps^{-1}, $\tau = 1$ ps, $T_f = 3000$ K, $\kappa = 500$ kJ/mol·nm^2 for distance variables and 1000 kJ/mol·rad^2 for χ_1) and their and corresponding probability density distributions overlaid with equilibrium distribution of the closed and open state (on the right). The raw data to plot the time evolution of the CVs was smoothed using a rolling function with a window size of 51.

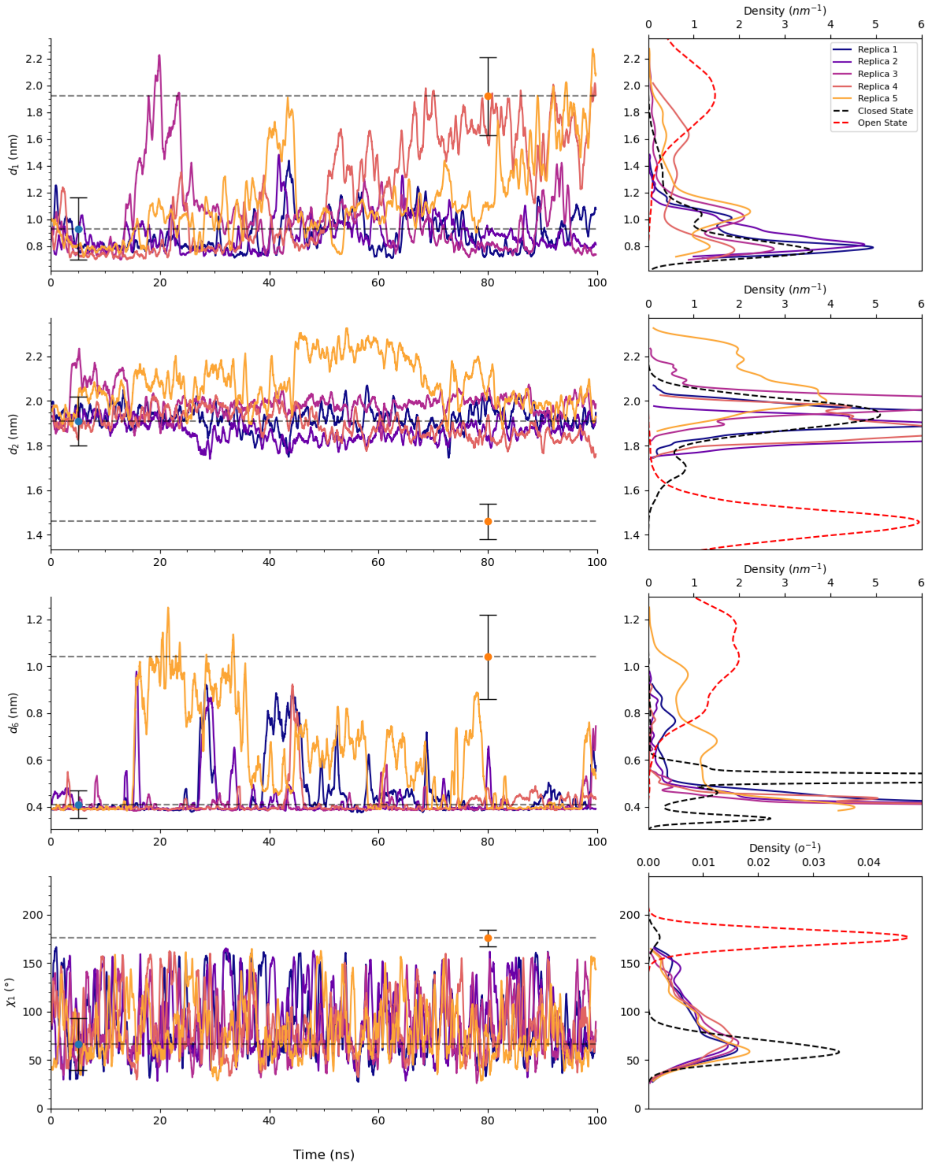

Figure S18: CVs d_1, d_2, d_6, and χ_1 v. time (on the left) from TAMD simulation set 2 (parameters $\gamma = 1$ ps^{-1}, $tau = 10$ ps, $T_f = 3000$ K, $\kappa = 500$ kJ/mol·nm^2 for distance variables and 1000 kJ/mol·rad^2 for χ_1) and their and corresponding probability density distributions overlaid with equilibrium distribution of the closed and open state (on the right). The raw data to plot the time evolution of the CVs was smoothed using a rolling function with a window size of 51.

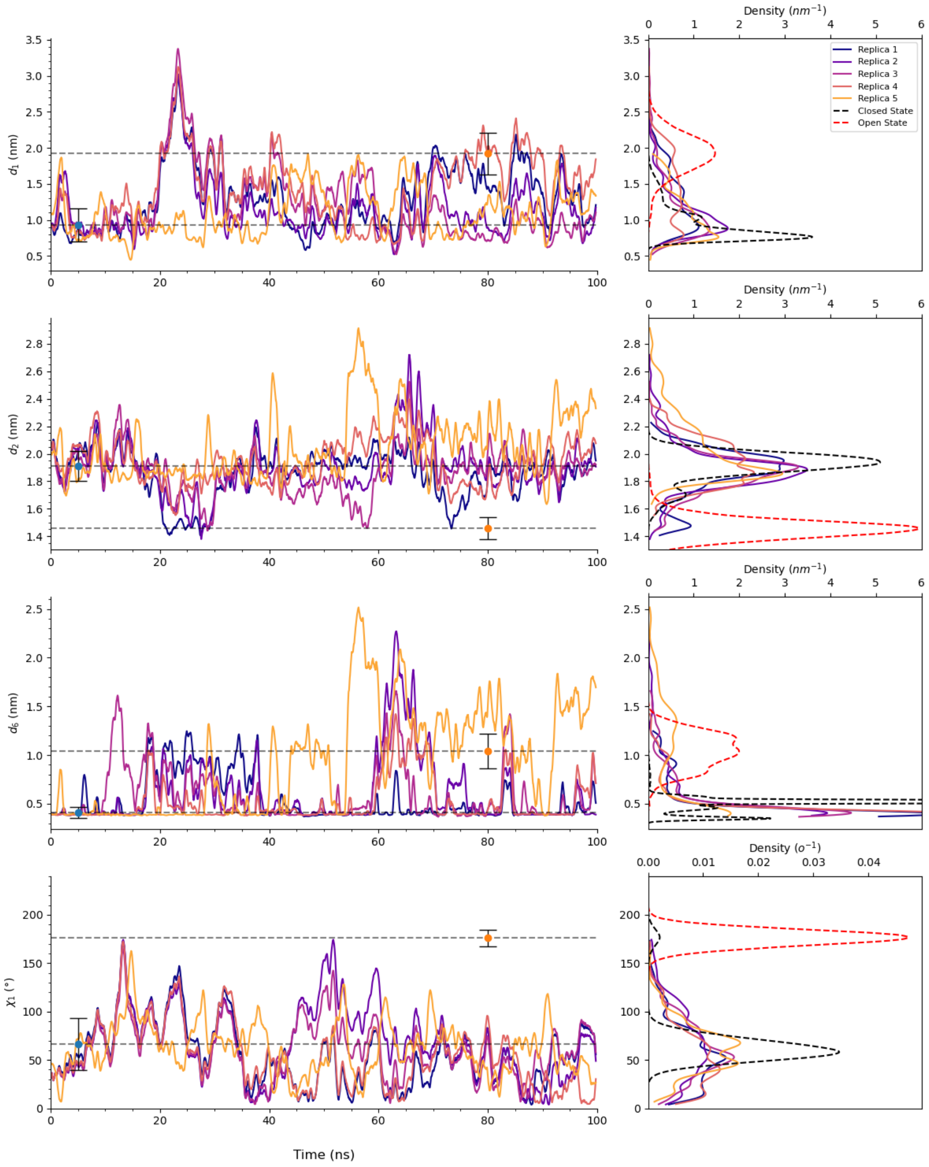

Figure S19: CVs d_1, d_2, d_6, and χ_1 v. time (on the left) from TAMD simulation set 3 (parameters $\gamma = 1$ ps^{-1}, $\tau = 100$ ps, $T_f = 3000$ K, $\kappa = 500$ kJ/mol·nm^2 for distance variables and 1000 kJ/mol·rad^2 for χ_1) and their and corresponding probability density distributions overlaid with equilibrium distribution of the closed and open state (on the right). The raw data to plot the time evolution of the CVs was smoothed using a rolling function with a window size of 51.

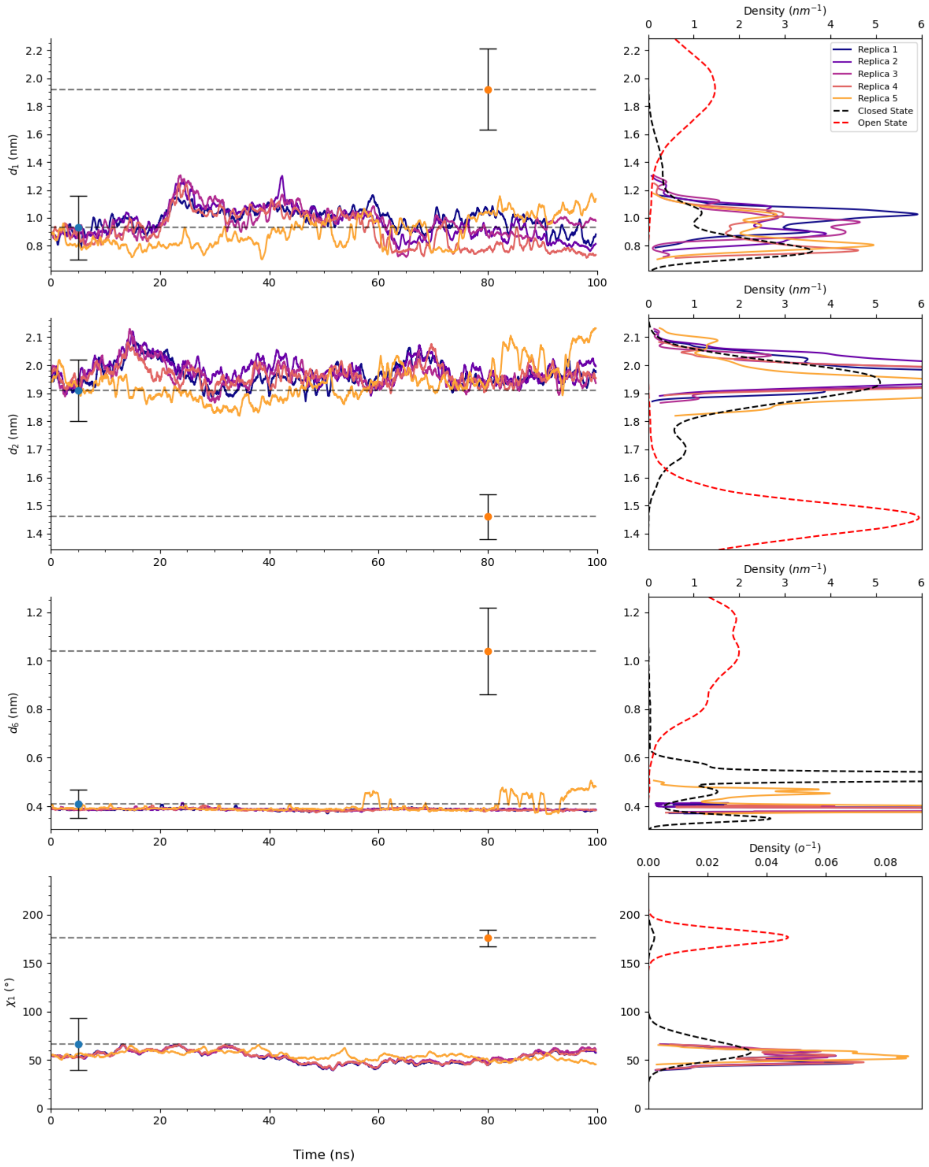

Figure S20: CVs d_1, d_2, d_6, and χ_1 v. time (on the left) from TAMD simulation set 4 (parameters $\gamma = 1$ ps^{-1}, $tau = 1000$ ps, $T_f = 3000$ K, $\kappa = 500$ kJ/mol·nm^2 for distance variables and 1000 kJ/mol·rad^2 for χ_1) and their and corresponding probability density distributions overlaid with equilibrium distribution of the closed and open state (on the right). The raw data to plot the time evolution of the CVs was smoothed using a rolling function with a window size of 51.

In sets 5 to 8 (Figs. S21–S23) we test increasing τ from 0.1 to 100 ps at a higher friction of 10 ps $^{-1}$ $\gamma = 10$ ps^{-1}. Our results portray that increasing fictitious friction to $\gamma = 10$ ps^{-1} significantly affects the sampling of the open state across different values of τ. At a low $\tau =$ 0.1 ps, all variables remain within the closed-state equilibrium, showing no transitions to the

open state. Increasing τ to 1 ps does not significantly alter this behavior. At $\tau = 10$ ps, the smaller CVs (d_6 and χ_1) begin to fluctuate and sample the open state, but the larger-scale motions of d_1 and d_2 remain restricted. Further increasing τ to 100 ps leads to overdamping, particularly for χ_1, which remains confined to closed-state values in all replicas. The same overdamping effect is observed for distance variables, indicating that a friction of 10 ps^{-1} at $\tau = 100$ ps suppresses motion and prevents effective sampling of the open-state equilibrium.

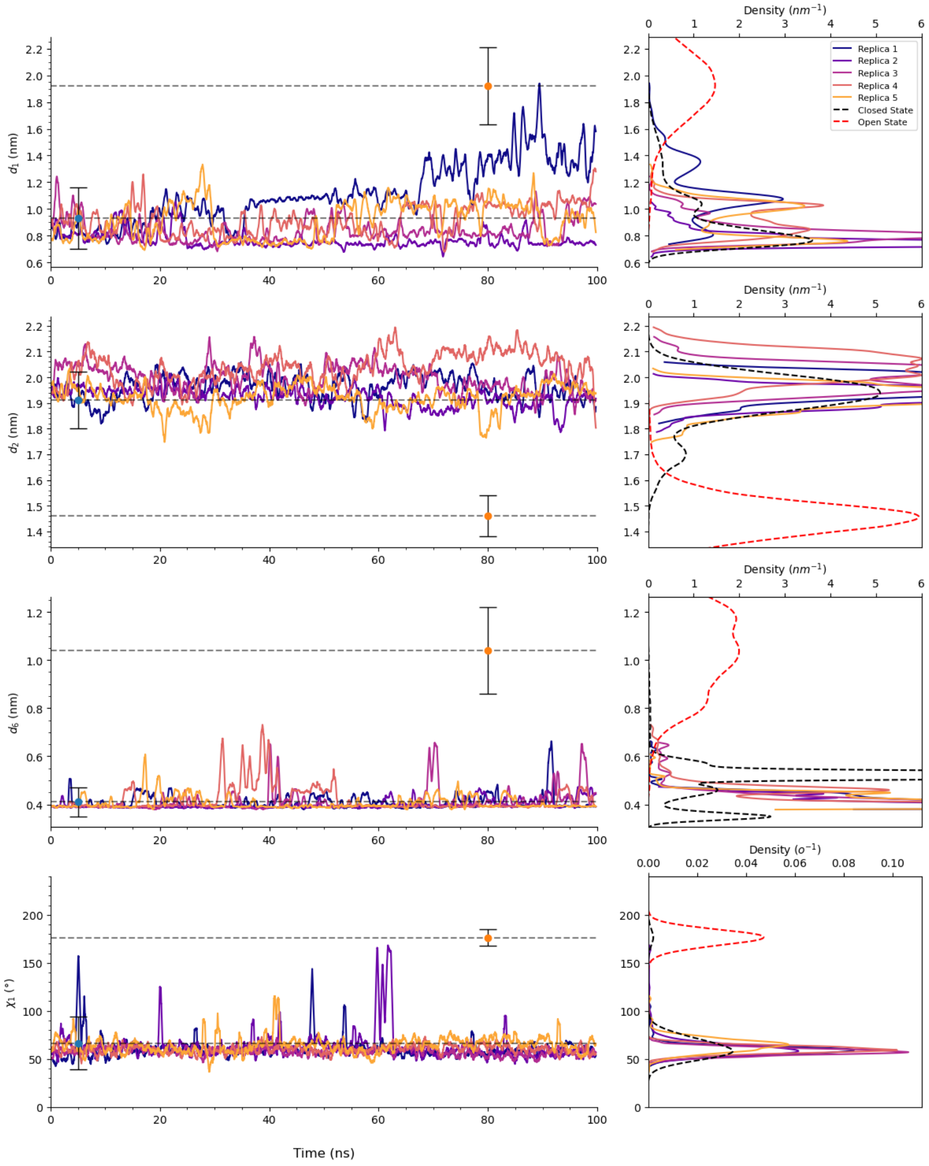

Figure S21: CVs d_1, d_2, d_6, and χ_1 v. time (on the left) from TAMD simulation set 5 (parameters γ = 10 ps^{-1}, tau = 0.1 ps, T_f = 3000 K, κ = 500 kJ/mol·nm^2 for distance variables and 1000 kJ/mol·rad^2 for χ_1) and their and corresponding probability density distributions overlaid with equilibrium distribution of the closed and open state (on the right). The raw data to plot the time evolution of the CVs was smoothed using a rolling function with a window size of 51.

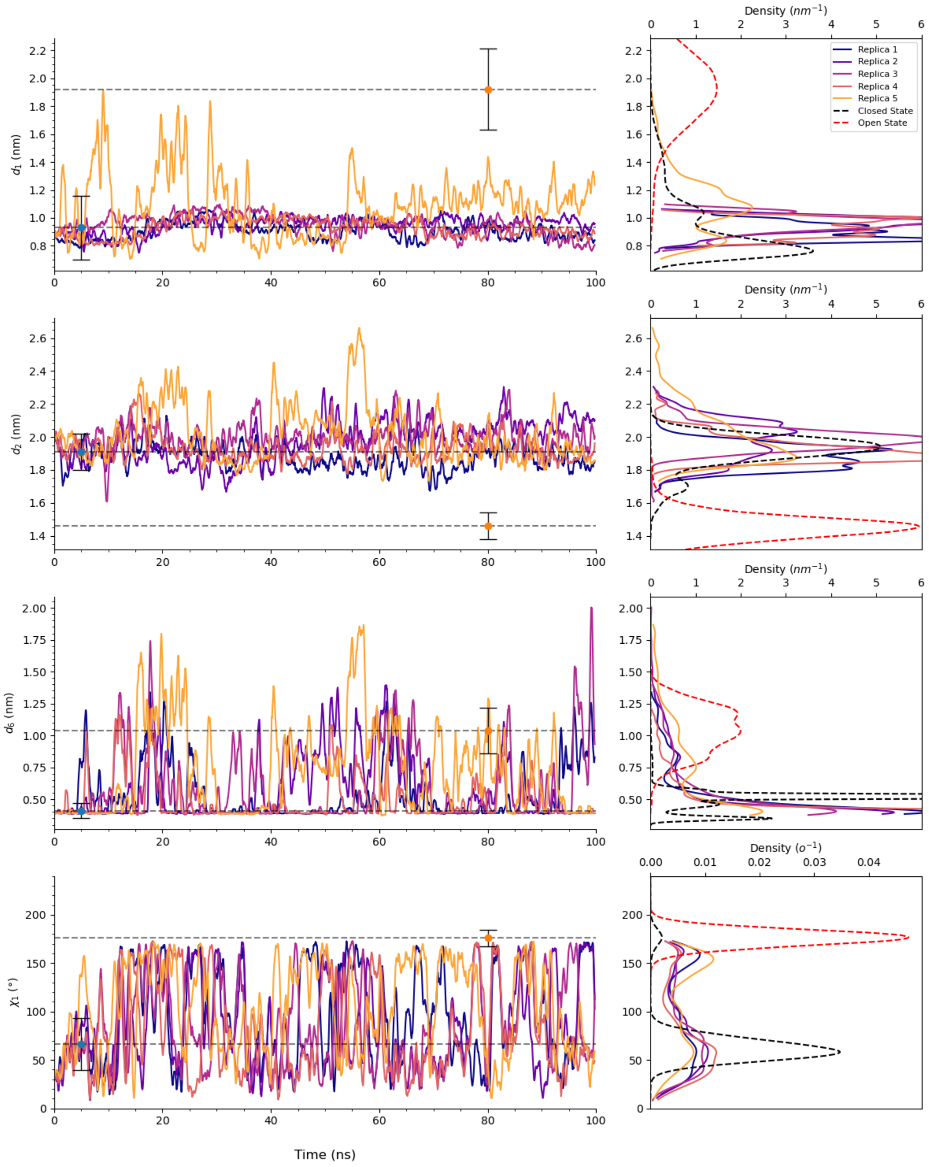

Figure S22: CVs d_1, d_2, d_6, and χ_1 v. time (on the left) from TAMD simulation set 7 (parameters $\gamma = 10$ ps^{-1}, $\tau = 10$ ps, $T_f = 3000$ K, $\kappa = 500$ kJ/mol·nm^2 for distance variables and 1000 kJ/mol·rad^2 for χ_1) and their and corresponding probability density distributions overlaid with equilibrium distribution of the closed and open state (on the right). The raw data to plot the time evolution of the CVs was smoothed using a rolling function with a window size of 51.

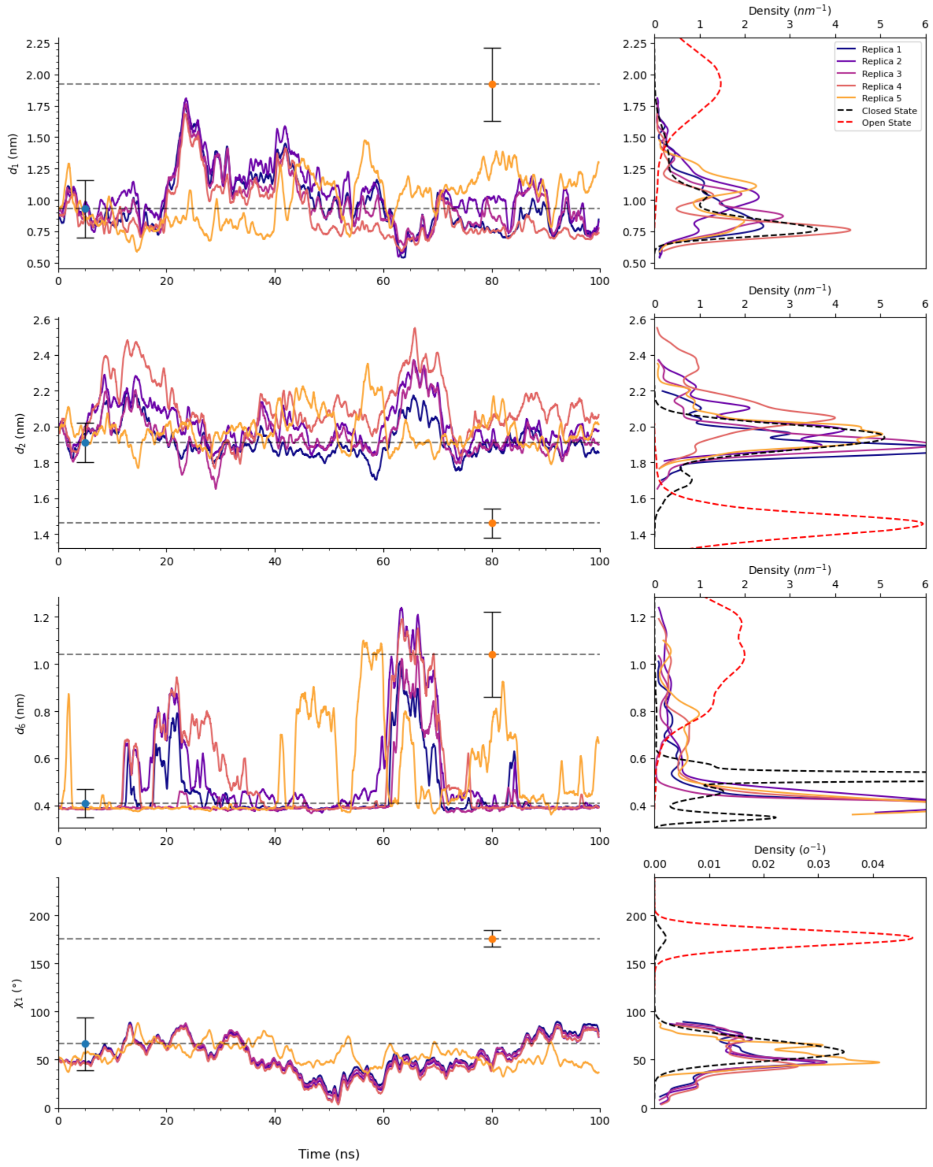

Figure S23: CVs d_1, d_2, d_6, and χ_1 v. time (on the left) from TAMD simulation set 8 (parameters $\gamma = 10$ ps^{-1}, $tau = 100$ ps, $T_f = 3000$ K, $\kappa = 500$ kJ/mol·nm^2 for distance variables and 1000 kJ/mol·rad^2 for χ_1) and their and corresponding probability density distributions overlaid with equilibrium distribution of the closed and open state (on the right). The raw data to plot the time evolution of the CVs was smoothed using a rolling function with a window size of 51.

The issue has been that d_2 does not fluctuate to smaller values, except when $\gamma = 1$ ps $^{-1}$ and $\tau = 100$ ps. Increasing γ from 1 to 10 ps^{-1} overdamps the CVs, so set 9 (Figs.S24) explores reducing γ from 1 to 0.1 ps^{-1} at $\tau = 100$ ps. We find that d_2 fluctuates less at fr $= 0.1$ ps^{-1}, occasionally reaching the open state but less frequently than at $\gamma = 1$ ps

$^{-1}$. Additionally, lower γ underdamps d_1, d_6, and χ_1, causing excessive oscillations without simultaneous sampling of the open state. We find that γ = 1 ps $^{-1}$ and τ = 100 ps at T_f = 3000 K allow all CVs to sample the open region, though not simultaneously. Further analysis is needed to understand how coupling strength and fictitious particle temperature affect their motion.

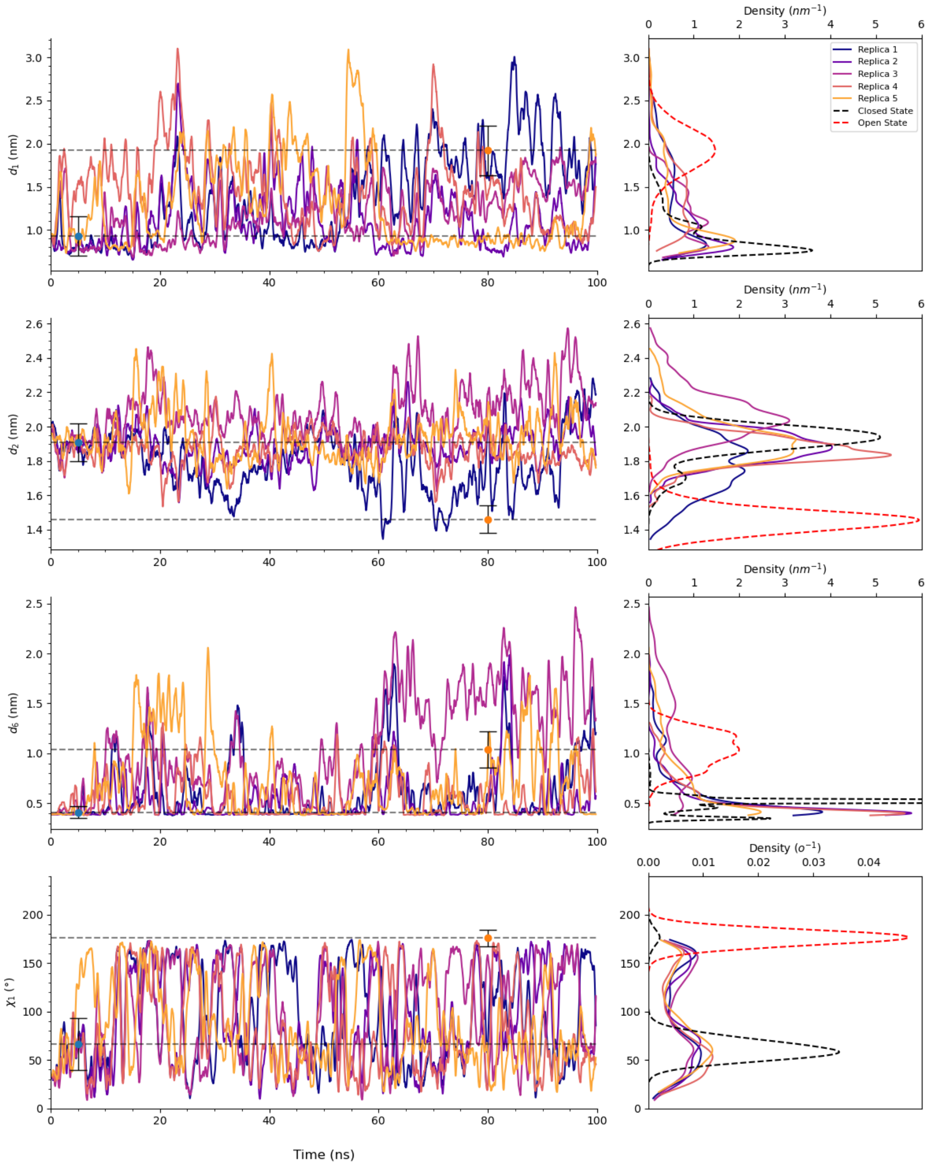

Figure S24: CVs d_1, d_2, d_6, and χ_1 v. time (on the left) from TAMD simulation set 9 (parameters $\gamma = 0.1$ ps^{-1}, $tau = 100$ ps, $T_f = 3000$ K, $\kappa = 500$ kJ/mol·nm^2 for distance variables and 1000 kJ/mol·rad^2 for χ_1) and their and corresponding probability density distributions overlaid with equilibrium distribution of the closed and open state (on the right). The raw data to plot the time evolution of the CVs was smoothed using a rolling function with a window size of 51.

In sets 10, 11, and 12 (Figs. S25–S27), we explore the effects of increasing the fictitious temperature for all or some variables. In set 10, raising the temperature to 6000 K for all four variables caused d_1, d_2, and d_6 to increase significantly, leading to dramatic denaturation of α_3 and displacement of α_1.

In set 11, T_f was increased to 6000 K only for d_2 and d_6, while d_1 and χ_1 remained at T_f of 3000 K. Despite this, d_6 reached large separations, while χ_1 unexpectedly fluctuated around the closed equilibrium in four out of five replicas, suggesting d_2 and d_6 may influence its behavior. Although d_2 occasionally decreased, it still did not sample the open state.

In set 12, temperatures for d_1 and d_2 (large domain motions) were kept at 3000 K, while d_6 and χ_1 (small-scale side-chain motions) were increased. This caused a salt bridge to break, unlatching α_3 from $alpha_1$ and leading d_2 to fluctuate at very high separations rather than decreasing. Meanwhile, χ_1 fluctuated but remained close to the closed region. These results suggest that increasing any variable's temperature above 3000 K leads to system instability. Future exploration should consider adjusting the coupling constant κ to facilitate equilibrium sampling of the open state where all variables fluctuate within their expected ranges.

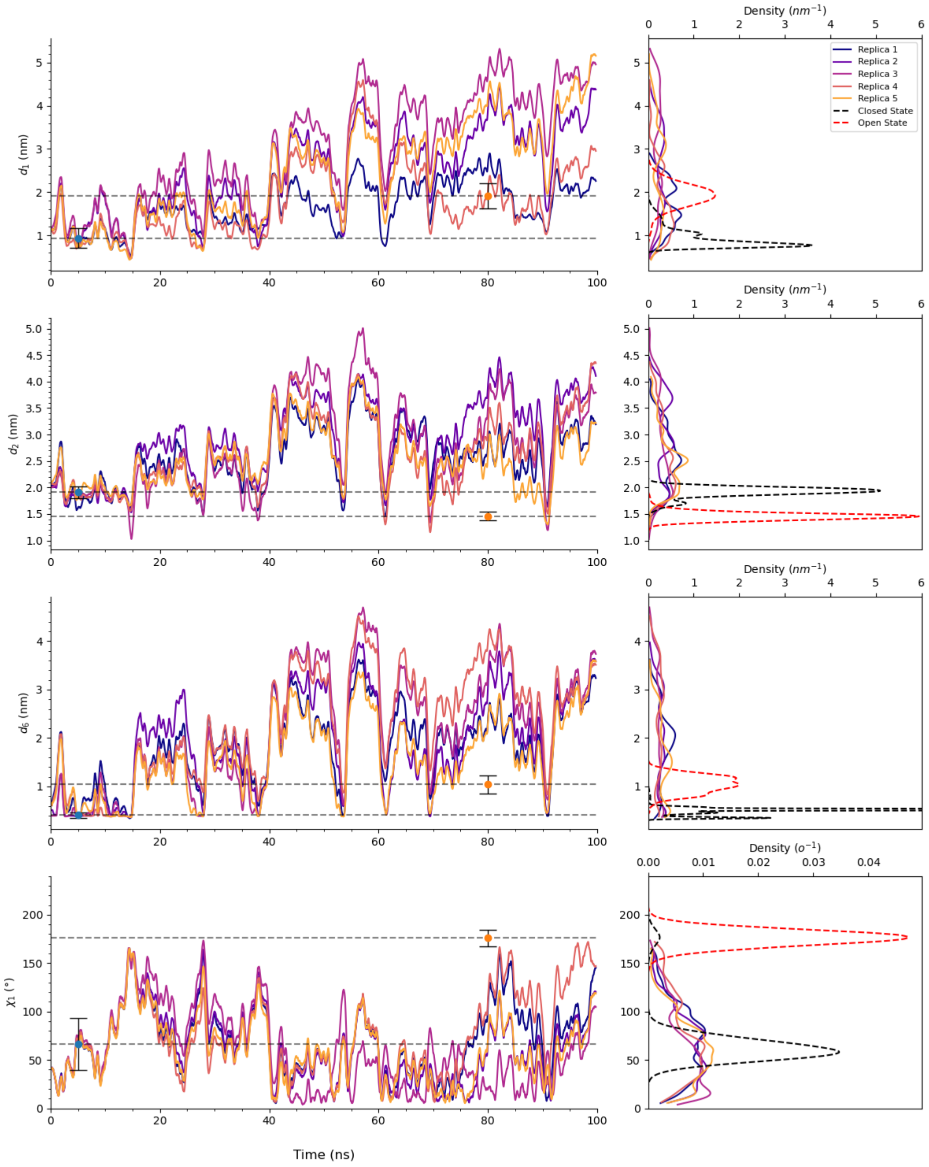

Figure S25: CVs d_1, d_2, d_6, and χ_1 v. time (on the left) from TAMD simulation set 10 (parameters $\gamma = 1$ ps^{-1}, $tau = 100$ ps, $T_f = 6000$ K, $\kappa = 500$ kJ/mol·nm^2 for distance variables and 1000 kJ/mol·rad^2 for χ_1) and their and corresponding probability density distributions overlaid with equilibrium distribution of the closed and open state (on the right). The raw data to plot the time evolution of the CVs was smoothed using a rolling function with a window size of 51.

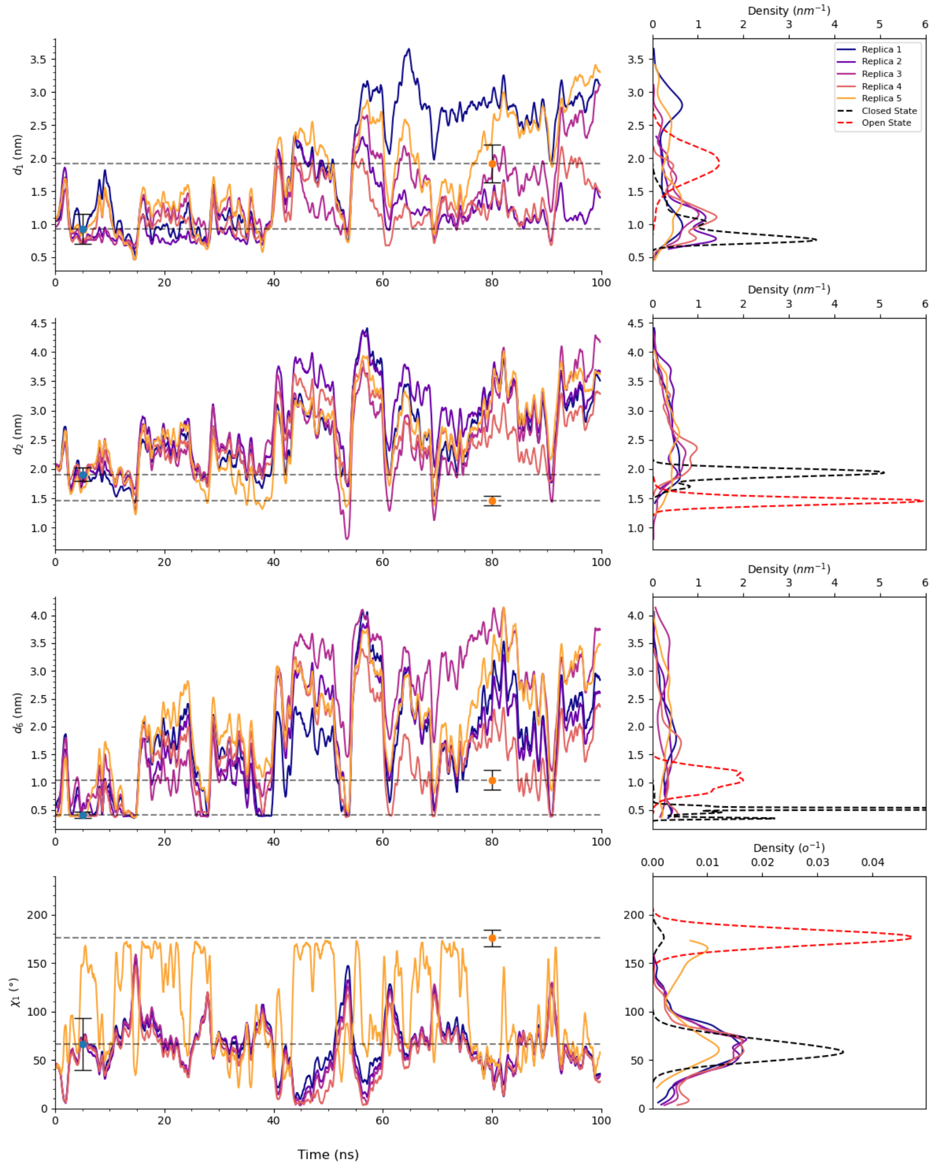

Figure S26: CVs d_1, d_2, d_6, and χ_1 v. time (on the left) from TAMD simulation set 11 (parameters $\gamma = 1$ ps^{-1}, $tau = 100$ ps, $T_f = 3000$ K for d_1 and χ_1 and 6000 K for d_2 and d_6, $\kappa = 500$ kJ/mol·nm^2 for distance variables and 1000 kJ/mol·rad^2 for χ_1) and their and corresponding probability density distributions overlaid with equilibrium distribution of the closed and open state (on the right). The raw data to plot the time evolution of the CVs was smoothed using a rolling function with a window size of 51.

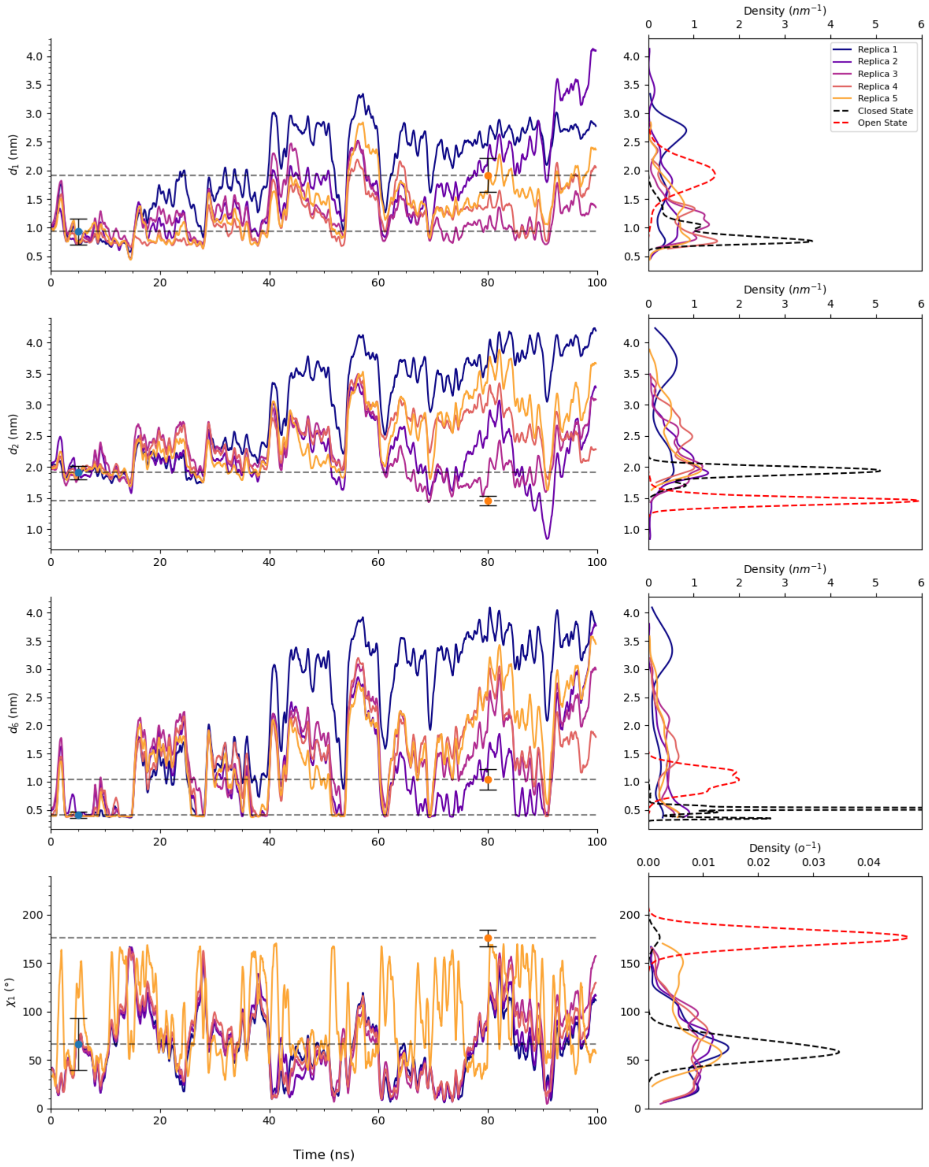

Figure S27: CVs d_1, d_2, d_6, and χ_1 v. time (on the left) from TAMD simulation set 12 (parameters γ = 1 ps^{-1}, tau = 100 ps, T_f = 3000 K for d_1 and d_2 and 6000 K for χ_1 and d_6, κ = 500 kJ/mol·nm^2 for distance variables and 1000 kJ/mol·rad^2 for χ_1) and their and corresponding probability density distributions overlaid with equilibrium distribution of the closed and open state (on the right). The raw data to plot the time evolution of the CVs was smoothed using a rolling function with a window size of 51.

In sets 13, 14, and 15 (Figs. S28 – 8CVs d_1, d_2, d_6, and χ_1 as a function of time (on the left) from TAMD simulation set 15 (parameters γ = 1 ps$^-1$, τ= 100 ps, T_f = 3000 K, κ=500 kJ/mol·nm^2 for distance variables and 100 kJ/mol·rad^2 for χ_1) and their and corresponding probability density distributions overlaid with equilibrium distribution of the closed and

open state (on the right). The raw data to plot the time evolution of the CVs was smoothed using a rolling function with a window size of 51.), we evaluate the effect of increasing and decreasing the coupling between the CVs and fictitious particle κ. In set 13 we increased kappa for all distance variables by double (to 1000 kJ/mol·nm^2) and kept χ_1 unchanged at 1000 kJ/mol·rad^2. In set 14 we decreased κ for all variables by a factor of 10, where κ of d_1, d_2, d_6 is 50 kJ/mol·nm^2 and 100 kJ/mol·rad^2 for χ_1. This led us to decrease κ for chi only to 100 kJ/mol·rad^2 in set 15 while keeping κ for all distance variables at 500 kJ/mol·nm^2. We found that κ of 500 kJ/mol·nm^2 for distance variables and 100 kJ/mol·rad^2 for χ_1 is optimal.

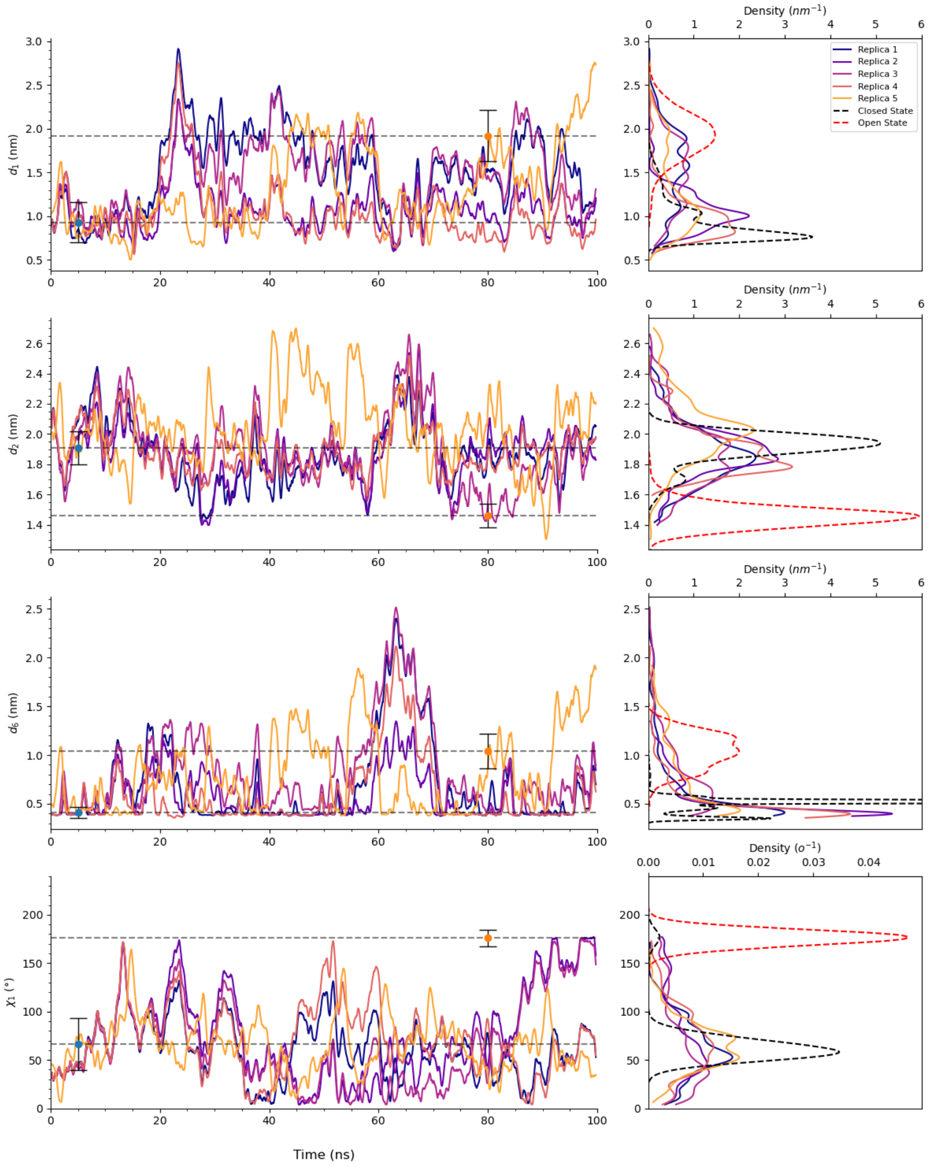

Figure S28: CVs d_1, d_2, d_6, and χ_1 v. time (on the left) from TAMD simulation set 13 (parameters $\gamma = 1$ ps^{-1}, $tau = 100$ ps, $T_f = 3000$ K, κ=1000 kJ/mol·nm^2 for all CVs) and their and corresponding probability density distributions overlaid with equilibrium distribution of the closed and open state (on the right). The raw data to plot the time evolution of the CVs was smoothed using a rolling function with a window size of 51.

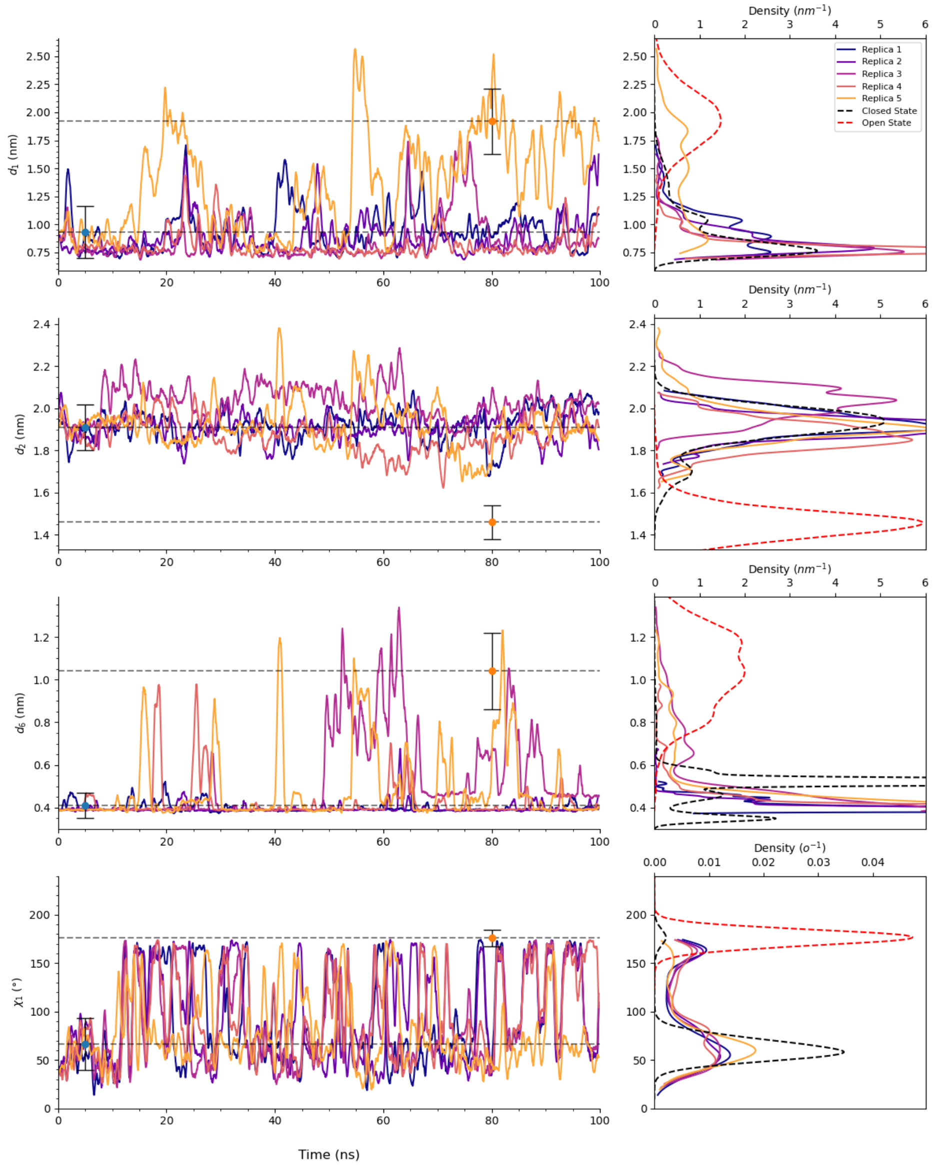

Figure S29: CVs d_1, d_2, d_6, and χ_1 v. time (on the left) from TAMD simulation set 14 (parameters $\gamma = 1$ ps^{-1}, $tau = 100$ ps, $T_f = 3000$ K, $\kappa = 50$ kJ/mol·nm^2 for distance variables and 100 kJ/mol·rad^2 for χ_1) and their and corresponding probability density distributions overlaid with equilibrium distribution of the closed and open state (on the right). The raw data to plot the time evolution of the CVs was smoothed using a rolling function with a window size of 51.

Using TAMD parameter combination in set 15, we perform TAMD simulation using CVs that failed to give conformational transition when biased in SMD simulations (Figs. S30 and S31).

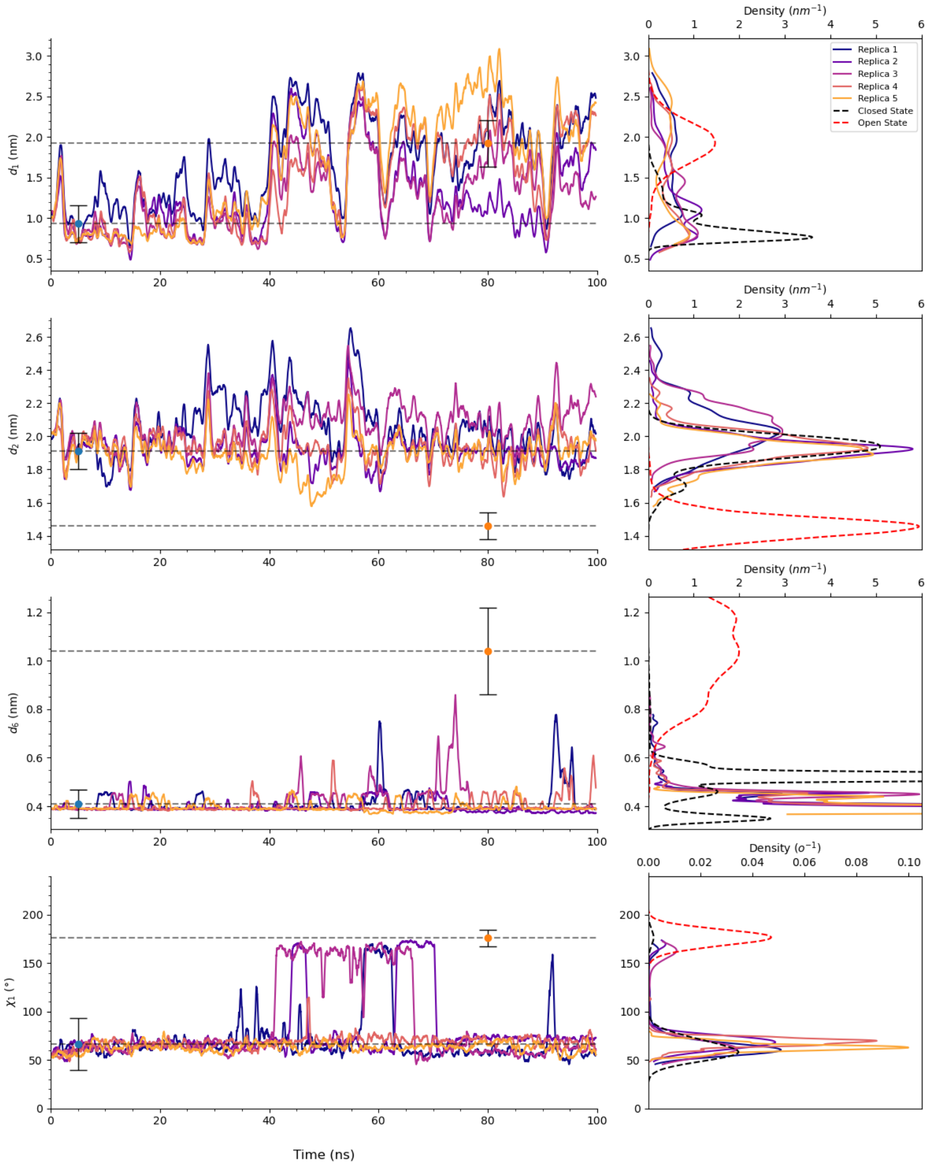

Figure S30: CVs d_1, d_2, d_6, and χ_1 v. time (on the left) from TAMD simulation set 15 (parameters $\gamma = 1$ ps^{-1}, $tau = 100$ ps, $T_f = 3000$ K, $\kappa = 500{\cdot}\text{nm}^2$ to couple d_1 and d_2 only) and their and corresponding probability density distributions overlaid with equilibrium distribution of the closed and open state (on the right). The raw data to plot the time evolution of the CVs was smoothed using a rolling function with a window size of 51.

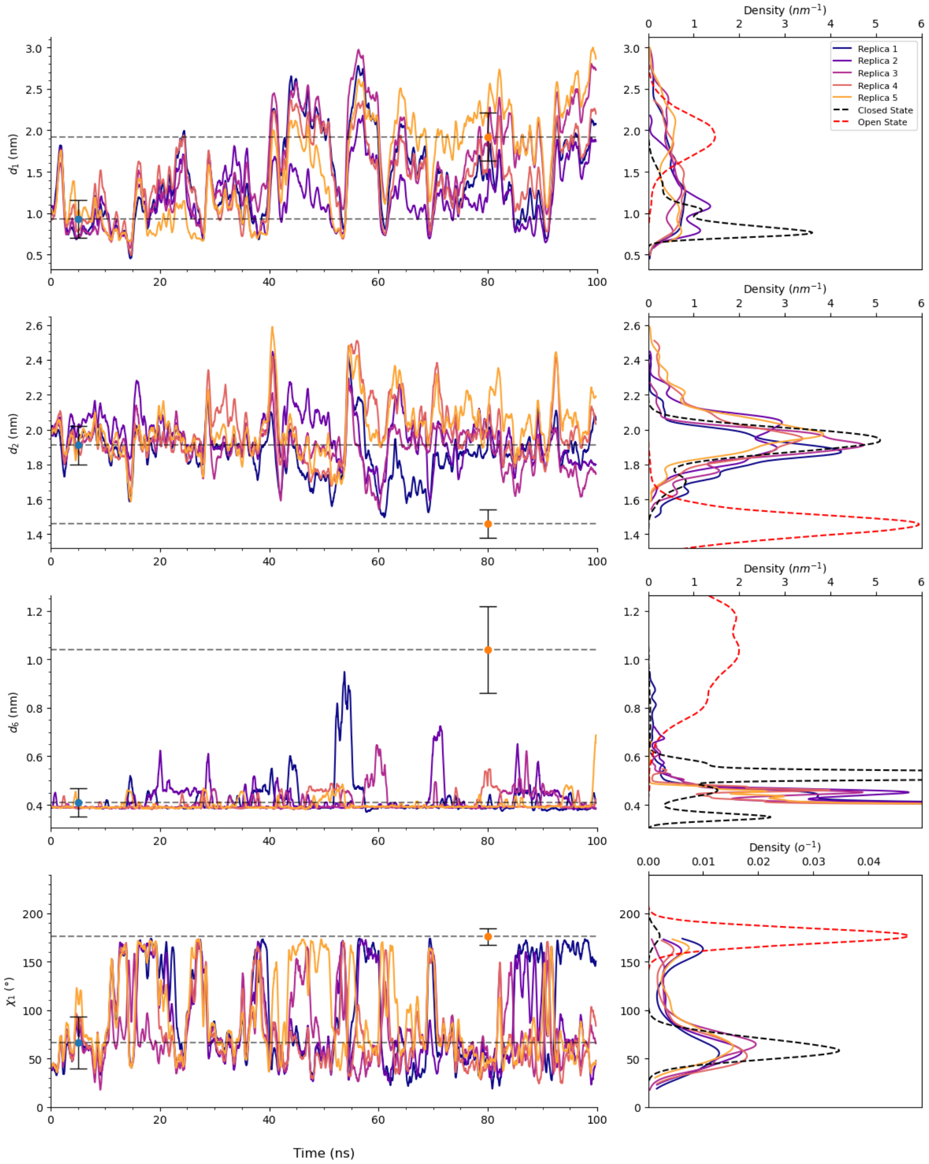

Figure S31: CVs d_1, d_2, d_6, and χ_1 v. time (on the left) from TAMD simulation set 15 (parameters $\gamma = 1$ ps^{-1}, $tau = 100$ ps, $T_f = 3000$ K, $\kappa = 500$ kJ/mol·nm^2 to bias d_1 and d_2, and $\kappa = 100$ kJ/mol·rad^2 to couple χ_1) and their and corresponding probability density distributions overlaid with equilibrium distribution of the closed and open state (on the right). The raw data to plot the time evolution of the CVs was smoothed using a rolling function with a window size of 51.
